# Spirulina Peptides Suppress UVB-Induced Skin Hyperpigmentation via Integrated Modulation of Melanogenesis and Inflammatory Pathways

**DOI:** 10.3390/antiox15020181

**Published:** 2026-01-30

**Authors:** Qiying Zeng, Kaiye Yang, Hongtao Gu, Changzhi Dong, Wei Zhou, Zhiyun Du

**Affiliations:** 1 School of Biomedical and Pharmaceutical Sciences, Guangdong University of Technology, Guangzhou 510006, China; 2 Infinitus (China) Company Ltd., Guangzhou 510405, China; 3 CNRS UMR 8251, INSERM ERL 1133, Unité de Biologie Fonctionnelle et Adaptative, Université Paris Cité, F-75013 Paris, France

**Keywords:** spirulina peptides, tyrosinase, melanin, pigmentation, signaling pathway

## Abstract

Background: Hyperpigmentation disorders lack effective therapies due to efficacy and safety limitations. *Spirulina*-derived peptides (SPs) show promises as anti-melanogenic agents, but their mechanisms remain unclear. Methods: SPs (<1 kDa, 3–6 amino acids) were isolated and assessed for tyrosinase inhibition, antioxidant, and anti-glycation activities. In vitro effects were tested in B16F10 cells; transcriptomic profiling used RNA sequencing. In vivo efficacy was evaluated in UVB-induced hyperpigmentation mouse models. Results: SPs exhibited mixed-type kinetic inhibition of tyrosinase along with strong antioxidant and anti-glycation activities. In vitro, SP suppressed melanin synthesis by directly inhibiting tyrosinase, downregulating the cAMP/PKA/CREB cascade, and activating the PI3K/Akt/GSK-3β pathway, resulting in reduced MITF and tyrosinase expression. Transcriptomic analysis revealed broad regulation of melanogenesis and inflammatory pathways. In vivo, topical SP treatment significantly reduced UVB-induced hyperpigmentation and skin inflammation, correlating with decreased CREB phosphorylation and tyrosinase expression. Conclusions: SP acts as a dual anti-melanogenic/anti-inflammatory agent through enzyme inhibition and signaling modulation, offering a novel therapeutic strategy for inflammation-associated hyperpigmentation.

## 1. Introduction

Melanin, a photoprotective pigment, governs skin color and protects against UV-induced DNA damage [1]. Melanogenesis, the biosynthetic pathway responsible for melanin production, occurs within melanocytes, specialized cells housing melanosomes—lysosome-related organelles [2,3]. This process is tightly regulated by the melanocortin 1 receptor (MC1R), which is activated by α-melanocyte-stimulating hormone (α-MSH). MC1R activation triggers cAMP-dependent signaling, thereby inducing the upregulation of microphthalmia-associated transcription factor (MITF) [4,5]. MITF orchestrates the expression of tyrosinase (TYR) and tyrosinase-related proteins (TRP-1/TRP-2), key enzymes critical for melanin synthesis [6]. Excessive melanin accumulation underlies hyperpigmentation disorders, such as melasma and solar lentigines, and exacerbates skin photoaging, which correlates with melanogenesis through oxidative stress and genomic instability [7].

The growing demand for skin-whitening products has spurred rigorous evaluation of the safety profiles of novel whitening agents relative to conventional counterparts. Hydroquinone, a widely used tyrosinase inhibitor, has raised significant safety concerns due to its cytotoxic and carcinogenic properties, with studies linking chronic use to ochronotic and mitochondrial DNA damage in melanocytes [8]. Similarly, kojic acid, despite its efficacy in melanogenesis inhibition, induces pro-oxidative effects and triggers keratinocyte apoptosis by disrupting redox homeostasis [9]. α-Arbutin, a natural glycoside, poses phototoxic risks under UV exposure through hydroquinone release, thereby exacerbating inflammatory responses and oxidative stress in epidermal cells [10]. Despite the therapeutic potential of natural compounds, their clinical translation remains constrained by poor solubility and bioavailability. For instance, resveratrol, a polyphenol with potent tyrosinase-inhibitory activity, exhibits limited epidermal penetration due to its inherent hydrophobic nature, necessitating nanoparticle encapsulation to enhance cutaneous delivery [11,12]. Likewise, despite its anti-melanogenic characteristics, curcumin undergoes rapid degradation and displays low stability in aqueous formulations, demanding structural modifications or lipid-based carriers [13,14]. These challenges underscore the need for innovative strategies to enhance the efficacy of natural whitening agents while ensuring safety and bioavailability.

Marine environments represent a vast and promising source of bioactive compounds. Peptides derived from marine organisms, in particular, are increasingly recognized for their potential in biomedical applications, aligning with sustainable utilization of aquatic resources. Recent research advances underscore the capacity of bioactive peptides to modulate critical cellular signaling pathways, including those regulating melanogenesis, oxidative stress, inflammation, and extracellular matrix homeostasis [15,16,17,18,19]. Bioactive peptides, characterized by unique structural features such as hydrophobicity and post-translational modification, exhibit superior bioavailability and target specificity compared to synthesized drugs [20,21]. Their ability to interact with cell surface receptors and intracellular signaling cascades positions them as compelling candidates for next-generation whitening agents while simultaneously promoting environmentally responsible marine resource exploitation [22,23].

*Spirulina*, a cyanobacterium of marine origin, has attracted significant scientific attention due to its rich profile of bioactive compounds, including polyunsaturated fatty acids, essential amino acids, and pigment complexes such as chlorophyll, carotenoids, phycocyanin, and phenolic derivatives [24,25,26]. *Spirulina* peptides (SPs), produced via enzymatic hydrolysis of algal proteins, typically possess molecular weights below 3 kDa. These peptides often feature hydrophobic domains that enhance transdermal permeability and have demonstrated low cytotoxicity compared to conventional tyrosinase inhibitors [27,28,29]. In the context of mitigating photoaging, marine biopeptides, notably SPs, have been reported to promote human fibroblast vitality by activating the EGFR/MAPK signaling pathway [30]. Furthermore, SPs downregulate the expression of MMP-1 and MMP-3 under UVB irradiation, suggesting protective effects against skin aging and photoaging [30]. While the short peptide P5 (SPSWY), isolated from microalgae phylogenetically related to SPs, exhibits potent tyrosinase inhibitory activity [31], the precise inhibitory mechanism and signaling pathways underlying SP-mediated tyrosinase suppression require further elucidation.

This study aimed to characterize the chemical composition of SPs and systematically investigate their anti-photoaging bioactivity. The inhibitory activity of SPs against tyrosinase activity was examined, including the kinetics of enzyme-catalyzed reactions at a molecular level. Furthermore, the capacity of SPs for free radical scavenging, inhibition of Advanced Glycation End-products (AGE) formation, and suppression of tyrosinase activity in B16F10 cells were evaluated, with an emphasis on elucidating the underlying in vitro signaling pathways. Finally, the protective efficacy of SP against UVB-induced photodamage was assessed in C57BL/6J mice, along with an exploration of the in vivo signaling mechanism. Collectively, these investigations aim to provide robust evidence supporting the potential of SP to mitigate skin photoaging, highlighting its promise for further application in dermatological treatment or cosmetic formulations derived from a valuable food resource.

## 2. Materials and Methods

### 2.1. Materials and Reagents

SP was provided by Guangzhou Kanglun Biotechnology Co., Ltd. (Guangzhou, Guangdong, China). Antibodies against TYR, TRP-1, TRP-2, AKT, p-AKT, MITF, CREB, p-CREB, p-GSK3β and p-GSK3β were purchased from Cell Signaling Technology Inc. (Beverly, MA, USA). Antibodies against GAPDH (Cat. No. ET1601-4) were purchased from HUABIO (Hangzhou, China). 1,1-Diphenyl-2-picrylhydrazyl radical (DPPH•), Diammonium 2,2′-azino-bis(3-ethylbenzothiazoline-6-sulfonate) (ABTS), Methylglyoxal, Bovine serum albumin (BSA), Methylglyoxal (MGO) were obtained from Sigma-Aldrich Chemical Co. (St. Louis, MO, USA). Sodium chloride (NaCl) and Potassium chloride (KCl) were acquired from Shanghai Yansu Technology Co., Ltd. (Shanghai, China; mall.shiyanjia.com). L-DOPA (Cat. No. T0848) and α-arbutin (Cat. No. T4911) were purchased from TargetMol Chemicals Inc. (Boston, MA, USA). Mouse melanoma cells (B16F10, Cat. No. CL-0319) were purchased from Wuhan Procell Life Science & Technology Co., Ltd. (Wuhan, China). 3-(4,5-dimethylthiazol-2-yl)-2,5-diphenyl tetrazolium bromide (MTT), Triton X-100, Dimethyl sulfoxide (DMSO), and PBS were obtained from Sigma-Aldrich Chemical Co. (St. Louis, MO, USA). Phenylmethylsulphonyl fluoride (PMSF, Cat. No. IP0280) and trypsin-EDTA (Cat. No. IE5420) were obtained from Beijing Solarbio Science & Technology Co., Ltd. (Beijing, China). Common cell culture reagents, such as the penicillin-streptomycin antibody mixture, L-glutamine, and Dulbecco’s modified Eagle’s medium (DMEM), were purchased from Gibco Company (Dublin, Ireland). Fetal bovine serum (FBS, Cat. No. 209111) was purchased from Wuxi NEST Biotechnology Co., Ltd. (Wuxi, China). Antibody diluents for primary and secondary applications were sourced from Santa Cruz Biotechnology Inc. (Dallas, TX, USA). The BCA Protein Colorimetric Assay Kit (Cat. No. E-BC-K318-M) was purchased from Elabscience Biotechnology Co., Ltd. (Wuhan, China). The cAMP-ELISA kit, Mouse IL-18 (Interleukin 18) ELISA Kit, Mouse IL-33 (Interleukin 33) ELISA Kit, the ELISA Kit for Mouse GM-CSF (Granulocyte-Macrophage Colony Stimulating Factor) and the ELISA Kit for Mouse Prostaglandin E2 (PGE-2) were acquired from Thermo Scientific (Waltham, MA, USA). All other general reagents and miscellaneous commercial grade labware were obtained from Merck & Co., Inc. (Darmstadt, Germany).

### 2.2. Preparation of Spirulina Peptides (SPs)

Arthrospira platensis was subjected to enzymatic hydrolysis with lysozyme (pH 7.0–7.5, 35 °C, 1–4 h), followed by ultrasonic disruption (200–400 W, 5–10 s pulses). The crude extract was centrifuged (8000–12,000 rpm, 10–20 min) to remove insoluble debris. The supernatant was further purified using ultrafiltration with a molecular weight cut-off (MWCO) of 3 kDa to remove macromolecules. The filtrate containing small peptides was collected and lyophilized to obtain the final SP powder. This standardized preparation process was carried out by Guangzhou Kanglun Biotechnology Co., Ltd. (Guangzhou, Guangdong, China) to ensure batch reproducibility.

**Figure 1:**
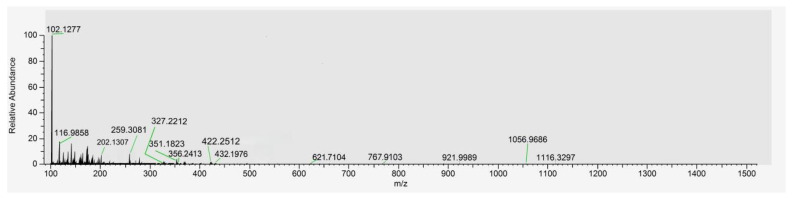
Total ion chromatogram of peptides from Spirulina-derived peptides (SPs).

To characterize the specific peptide sequences within SP, a sample was analyzed via SDS-PAGE (12% gel). Target gel bands (or the purified SP solution) were processed and identified using LC-MS/MS. The total ion chromatogram and identified sequences serve as the compositional fingerprint for SP standardization (Figure 1 and Table 1).

### 2.3. Determination of Molecular Weight Distribution

Before analysis, the SP was treated with reductive alkylation and then desalted using a self-priming desalting column. Following this, the alkylated and desalted SP sample was isolated using ultra-high-performance liquid chromatography (UHPLC) (Ultimate 3000; Thermo Fisher Scientific Inc., Waltham, MA, USA) with an Acclaim Pep Map RPLC C18AQ (Φ 75 µm× 150 mm) column (Dionex TM, Thermo Scientific). 0.1% formic acid (FA) (solvent A) and 0.1% FA/80% ACN (acetonitrile, solvent B) made up the mobile phase. The UV detector’s wavelength was 220 nm, the flow rate was 600 μL/min, and the gradient condition was 6% B to 40% B in 75 min (maintained at 95% B for 3 min). The molecular mass distribution of SPs was ultimately identified using electrospray ionization mass spectrometry and tandem mass spectrometry (ESI-MS/MS) in a positive ion mode.

### 2.4. Major Peptide Sequence Analysis of SPs

Peptide sequences were identified using electrospray ionization mass spectrometry and tandem mass spectrometry (ESI-MS/MS) in positive ion mode. After chromatography, ESI-MS/MS was performed with a Q ExactiveTM triple quadrupole device (Thermo Fisher Scientific, Waltham, MA, USA) with an ESI source attached. The sequences of distinctive peptides were identified by examining and contrasting secondary fragments of peptides from the collision-induced dissociation spectrum of the protonated molecule [M + H]^+^ in the UniProt database. The operational conditions of the mass spectrometer were followed as outlined below: Resolution: 75,000, AGC target: 1 × 10^5^, maximum IT: 60 ms, Top N: 20, NCE/stepped NCE: 27, Scan range: 50 to 1500 *m*/*z*.

### 2.5. Amino Acid Composition of SPs

The amino acid concentration and composition of SPs were examined using an automated analyzer. Initially, a hydrolysis tube containing phenol was filled with 10 mL of 5 mol/L HCl. Subsequently, the mixture underwent vacuum treatment and was purged with nitrogen before hydrolysis at 110 °C for 24 h. After cooling, the standard amino acid samples and filtrate were added to the analyzer. The concentrations of amino acids in the sample were determined by referencing the standard curve.

### 2.6. Determination of TYR Inhibition

The TYR-inhibitory activity was assessed utilizing a 96-well plate, adhering to a modified methodology inspired by the approach outlined by Masuda et al. [32]. TYR was prepared using a concentration of 100 U/mL in a 0.05 M sodium phosphate buffer at pH 6.8 [33]. The concentrations of SPs in the samples were 0, 0.0625, 0.125, 0.25, 0.5, 1, and 2 mg/mL. The wells have been allocated for the subsequent mixtures: 120 μL of 0.05 M sodium phosphate buffer (pH 6.8) and 40 μL of TYR were used as the control (no sample); 160 μL of the buffer was used as the blank (no sample and TYR); Prepare 80 μL of the buffer, 40 μL of TYR, and 40 μL of the sample. For the blank sample (excluding TYR), combine 120 μL of the buffer with 40 μL of the sample solution. The contents of each well were meticulously mixed with a microplate mixer and incubated at room temperature for 10 min. Each well was then introduced to 40 μL of 2.5 mM L-DOPA, formulated in the identical buffer. The solution was subsequently incubated at room temperature for an additional 2 min. The absorbance (A) at 475 nm was determined utilizing a microplate reader (ELX800, BioTek Industries, Winooski, VT,  USA). The percentage of TYR-inhibitory activity was determined using the following method:

$$\mathrm{TYR}\;\mathrm{inhibitory}\;\mathrm{activity}\;\left(\%\right)=\frac{\left(A_{\mathrm{control}}-A_{\mathrm{blank}}\right)-\left(A_{\mathrm{sample}}-A_{\mathrm{blank}\;\mathrm{sample}}\right)}{A_{\mathrm{control}}-A_{\mathrm{blank}}}\times 100\%$$

The inhibitory activity at different concentrations was plotted using Prism Graphpad (version 9.5.1, GraphPad Software, San Diego, CA, USA) and the IC_50_ value was determined from the fitted dose–response curve.

### 2.7. Inhibition Mechanism and Kinetics of TYR

The direct effects of SP on the diphenolase activity of TYR were assessed utilizing mushroom TYR with a constant concentration of L-DOPA as the substrate. In summary, 50 µL of SP (0.0625, 0.125, 0.25, 0.5, 1, and 2 mg/mL) was introduced into the wells of a 96-well microplate, followed by adding 50 µL of 15 mM L-DOPA. After adding 50 µL of mushroom TYR (12.5, 25, 50, 100, and 200 U/mL), the reaction was started and incubated for 10 min at 37 °C. Absorbance was recorded at a wavelength of 490 nm utilizing an enzyme-labeled instrument (Multiskan SkyHigh, Thermo Scientific, USA). The inhibition type exerted by SP on TYR was evaluated by measuring absorbance changes during the enzymatic reaction at various concentrations of SP.

The kinetic analysis of TYR inhibition was conducted as previously outlined, with minor modifications implemented [33]. TYR (50 U/mL) and SP (0.125, 0.25, 0.5, 1, 1.5, 2 mg/mL) in the primary reaction mixture were incubated at 37 °C for 10 min. Incubations without substrate were used as controls. Aliquots of the initial mixtures were transferred to a secondary reaction mixture containing L-DOPA (4, 6, 8, 10, 15 mM) in sodium phosphate buffer (pH 6.8). The reciprocal of the substrate concentration (1/[S]) was plotted against the reciprocal of the reaction rate (1/V) to construct Lineweaver–Burk plots. These plots were used to determine the inhibition type and associated kinetic parameters of SP on TYR.

### 2.8. Determination of Copper-Chelating Activity

The copper chelation activity of SP at various concentrations was determined using the modified method developed by Kubglomsong S et al. [34]. The concentrations of SP in the samples were 0, 0.0625, 0.125, 0.25, 0.5, 1, and 2 mg/mL. In particular, 10 μL of a 4 mM pyrocatechol violet solution made in the same buffer, 10 μL of a 1 μg/μL CuSO_4_·5H_2_O solution, and 100 μL of the sample’s SP were mixed with 100 μL of a 50 mM sodium acetate buffer (pH 6.0). The absence of the blue color was tracked by assessing the absorbance at 632 nm with a microplate reader (ELX800, BioTek Industries, USA). Water was utilized as a control instead of a sample. The calculation of the percentage of copper-chelating activity was performed based on the absorbance (A) measured at 632 nm using the following formula:

$$\mathrm{Copper}\text{-}\mathrm{Chelating}\;\mathrm{Activity}\left(\%\right)\;=\;\frac{A_{\mathrm{control}}-A_{\mathrm{sample}\;}}{A_{\mathrm{control}}}\times 100\%$$

The copper-chelating activity at different concentrations was plotted using Prism GraphPad and the IC_50_ value was determined from the fitted dose–response curve.

### 2.9. DPPH Radical Scavenging Activity

SP’s DPPH radical scavenging activity was evaluated using the method developed by Ma Y et al. [35] with slight modifications. The concentration gradients of glutathione (GSH) and the sample were 0.0625, 0.125, 0.25, 0.5, 1, and 2 mg/mL, respectively, in test solutions. Following this, 10 µL of the sample solution was mixed with 190 µL of DPPH solution (0.1 mg/mL in ethanol) and incubated for 30 min at room temperature without light. The absorbance at 517 nm was determined utilizing an enzyme-labeled instrument (Multiskan SkyHigh, Thermo Scientific, USA). Ethanol was utilized as a control instead of the DPPH solution, whereas ethanol without the sample functioned as a blank. GSH served as positive control. The following formula was used to determine the samples’ DPPH radical scavenging activity:

$$\mathrm{DPPH}\;\mathrm{Radical}\;\mathrm{Scavenging}\;\mathrm{Activity}\;\left(\%\right)\;=\;1-\frac{A_{\mathrm{sample}}-A_{\mathrm{control}}}{A_{\mathrm{blank}}}\;\times \;100\%$$

The DPPH radical scavenging activity at different concentrations was plotted using Prism GraphPad and the IC_50_ value was determined from the fitted dose–response curve.

### 2.10. ABTS Radical Scavenging Activity

SP’s ABTS radical scavenging activity was assessed following the methodology outlined by Ma Y et al., with slight modifications implemented [35]. 0.0625, 0.125, 0.25, 0.5, 1, and 2 mg/mL concentration gradients of GSH and the sample were added to the test solution. The ABTS solution was made using a stock solution with 7 mmol/L ABTS and 2.5 mmol/L potassium persulfate. The mixture was maintained in a dark environment at room temperature for 15 h to facilitate the formation of ABTS+ cations. The ABTS working solution was diluted in distilled water to absorb approximately 0.70 ± 0.02 at a wavelength of 734 nm. Following this, 1 mL of the sample was combined with 2 mL of the prepared ABTS working solution and incubated for ten min at room temperature without light. The absorbance was recorded at a wavelength of 734 nm, utilizing distilled water as a blank rather than the sample itself; GSH functioned as a positive control. GSH acted as positive control. The scavenging activity against ABTS radicals demonstrated by the samples was determined using the following equation:

$$\mathrm{ABTS}\;\mathrm{radical}\;\mathrm{scavenging}\;\mathrm{activity}\left(\%\right)\;=\;\frac{A_{\mathrm{blank}}-A_{\mathrm{sample}}}{A_{\mathrm{blank}}}\;\times \;100\%$$

The ABTS radical scavenging activity at different concentrations was plotted using Prism GraphPad and the IC_50_ value was determined from the fitted dose–response curve.

### 2.11. Inhibiting the Formulation of AGEs

The capacity of SP to prevent the formation of AGEs was evaluated using the BSA-MGO model [36]. A solution of 20 mL BSA (20 mg/mL) and 1 mL MGO (20 mmol/L) was made with 0.2 M, pH 7.4 PBS that included 0.02% sodium azide. Transfer 280 microliters of the MGO-BSA mixture to a black microplate. Next, add 20 μL of each of the following samples: the positive control medication aminoguanidine (AG) and SP at concentrations of 0.0625, 0.125, 0.25, 0.5, 1, and 2 mg/mL. For 24 h, the combinations were left to glycosylate at 37 °C in the dark. A Hitachi F-4700 spectrofluorometer (Hitachi, Tokyo, Japan) was used to measure the fluorescence intensity. The excitation and emission wavelengths were 360 and 470 nm, respectively. The following formula was used to determine the percentage inhibition of the production of advanced glycation end products (AGEs):

$$\mathrm{Inhibiting}\;\mathrm{the}\;\mathrm{formulation}\;\mathrm{of}\;\mathrm{AGEs}\left(\%\right)\;=\;1-\frac{{\mathrm{FL}}_{\mathrm{sample}}-{\mathrm{FL}}_{\mathrm{control}}}{{\mathrm{FL}}_{\mathrm{blank}}-{\mathrm{FL}}_{\mathrm{blank}\;\mathrm{control}}}\;\times \;100\%$$

FL sample is the group’s fluorescence intensity, comprising AG or SP samples and MGO. Control fluorescence intensity without MGO is denoted by FL_(control). FL_blank is the fluorescence intensity of blank control without samples and MGO, while FL_blank (blank control) is the fluorescence intensity of MGO alone.

### 2.12. Cell Culture

At 37 °C with 5% CO_2_, B16F10 cells were cultivated in DMEM media supplemented with 10% fetal bovine serum (FBS) and 1% penicillin/streptomycin.

### 2.13. Cell Viability Assay

The viability of B16F10 melanoma cells following SP treatment was evaluated using the MTT assay, as described by Ha et al. [37]. B16F10 cells were seeded in 96-well plates at a density of 5 × 10^4^ cells/mL and allowed to adhere. The cells were then treated with various concentrations of SP (0.125–4 mg/mL) or the positive control α-arbutin (0.25 mg/mL) for 24, 48, or 72 h. α-arbutin was selected as the positive control due to its established efficacy as a tyrosinase inhibitor [38,39]. At the end of the treatment period, 10 µL of MTT solution was added directly to each well, and the cells were incubated for 3 h at 37 °C to allow the formation of formazan crystals. Following this incubation, the supernatant was cautiously aspirated from each well to avoid disturbing the crystals. Subsequently, 100 µL of DMSO was added to each well to dissolve the purple formazan crystals. Finally, the absorbance was measured at 570 nm using a SpectraMax190 plate reader (Molecular Devices Corporation, San Jose, CA, USA). The results were expressed as a percentage of the control group.

### 2.14. Measurement of Cellular Melanin Content

The cellular melanin content of B16F10 cells was assessed with a BCA protein assay kit, as described by Han JH et al. [40]. Cells were seeded in 6-well plates at a density of 1.5 × 10^5^ cells per well and incubated for 24 h. B16F10 cells were treated with various concentrations of SP (0.03125–0.75 mg/mL) or α-arbutin (0.25 mg/mL) for 48 h. Following the incubation period, the cells were washed with PBS and then lysed in 170 μL of 1 M NaOH containing 10% DMSO for 1 h at a temperature of 90 °C. The relative melanin content was quantified by measuring absorbance at 405 nm using an ELISA. Melanin levels were calculated based on a standard curve derived from synthetic melanin. Protein concentrations in the cell extracts were determined by utilizing a BCA protein assay kit. Melanin production was expressed as the total melanin content per unit of protein in the cell extract.

### 2.15. Measurement of Cellular TYR Activity

The cellular TYR activity of B16F10 cells was assessed with L-dihydroxyphenylalanine (L-DOPA), as described by Han et al. [40]. Cells were seeded in 96-well plates at a density of 5 × 10^4^ cells per well and incubated for 24 h. Following this, the cells were treated with various concentrations of SP (0.03125–0.75 mg/mL) or α-arbutin (0.25 mg/mL) for an additional 48 h. Concurrently, the control group was maintained in DMEM without SP supplementation. After treatment, the medium was removed, and the cells were washed twice with PBS. Subsequently, 100 μL of 1% Triton X-100 (containing 1 mM PMSF) was added to each well. The cells were then lysed through multiple freeze–thaw cycles (3–5 times). The lysates were centrifuged to remove cell debris, and the supernatant was collected. For the activity assay, 50 μL of the supernatant and 50 μL of a solution containing 0.1% L-DOPA were added to wells in a new 96-well plate. The mixtures were incubated at 37 °C for one hour. After incubation, the concentration of dopachrome was quantified by measuring absorbance at a wavelength of 475 nm. The TYR activity observed in the control group represented an activity level of 100%.

### 2.16. Measurement of Intracellular cAMP Concentration

The intracellular cAMP concentration of B16F10 cells was assessed with a cAMP assay described by Han JH et al. [40]. Cells were seeded in 96-well plates at a density of 5 × 10^4^ cells per well and incubated for 24 h. Following this incubation period, the cells were treated with various concentrations of SP (0.03125–0.75 mg/mL) or α-arbutin (0.25 mg/mL) for an additional 48 h. Harvest the samples at the specified time points (1, 3, 6, and 9 h) and lyse them in lysis buffer. Cell lysates were incubated in specific rabbit antibody-coated microplates for 18 h at 4 °C. Following incubation, the wells were thoroughly washed, and cAMP conjugate was added to all wells. After a further incubation period of 90 min on an orbital shaker at room temperature, absorbance was measured using an ELISA reader set to detect wavelengths at 405 nm.

### 2.17. Western Blot Analysis in B16F10 Cells

B16F10 cells were treated with SP (0–0.5 mg/mL) or α-arbutin (0.25 mg/mL) for 48 h. The cells were then harvested with 1 mL of cold PBS and lysed with lysis buffer (100–300 μL) containing protease inhibitors (200 mM AEBSF, 30 μM Aprotinin, 13 mM Bestatin, 1.4 mM E64, and 1 mM Leupeptin in DMSO). The lysed samples were centrifuged at 16,000× *g* for 10 min at 4 °C, and the total protein concentration was determined using the BCA protein assay reagent. The samples were stored at −80 °C for subsequent analysis. Equal amounts of protein were separated by SDS-PAGE and transferred onto Polyvinylidene Difluoride (PVDF) membranes. Subsequently, the membranes were blocked with 3% (*w*/*v*) BSA for a minimum of 30 min at room temperature. After washing, the membranes were incubated with primary antibodies overnight at 4 °C. The antibodies used were as follows: anti-GAPDH (1:3000), anti-TYR (1:1000), anti-TRP-1 (1:1000), anti-TRP-2 (1:1000), anti-MITF (1:500), anti-CREB (1:1000), anti-p-CREB (1:1000), anti-AKT (1:1000), anti-p-AKT (1:1000), anti-GSK3β (1:1000), and anti-p-GSK3β (1:1000). After rinsing three times with TBST (5 min per wash), the membranes were incubated with HRP-conjugated secondary antibodies for 2 h at room temperature (25 °C). The protein bands were detected using enhanced chemiluminescence (ECL), and densitometric analysis was performed using ImageJ software (version 1.53k; National Institutes of Health, Bethesda, MD, USA).

### 2.18. Real-Time Fluorescence Quantitative PCR (Q-PCR)

To analyze the expression of the TYR, TRP-1, TRP-2, and MITF mRNA, we performed Q-PCR. The cells exposed to SP or α-arbutin for 48 h were harvested, and total RNA was extracted using a Trizol reagent. The primer pairs used for the Q-PCR analysis is listed in the table below. For the real-time PCR step, 2 μL of cDNA was amplified using TB Green Premix Ex Taq. The PCR reaction consisted of a denaturation step at 95 °C for 30 s, followed by 40 cycles of denaturation at 95 °C for 15 s and annealing at 60 °C for 30 s. The dissolve program included the following steps: 95 °C for 15 s, 60 °C for 60 s, and 95 °C for 1 s. Each sample was analyzed in triplicate. Relative gene expression levels were calculated using the 2^−ΔΔCt^ method.

### 2.19. RNA Extraction and Sequencing

B16F10 Cells were seeded in 6-well plates at a density of 1.5 × 10^5^ cells per well and incubated for 24 h. Cells were then treated with various concentrations of SP (0.125, 0.25, and 0.5 mg/mL) or α-arbutin (0.25 mg/mL) for 48 h. The control group received no treatment. Three independent biological replicates were prepared and sequenced separately for each group to ensure statistical validity.

All RNA extraction, cDNA library generation, and RNA sequencing (RNA-seq) were performed by Banian Medical Technology Co., Ltd. (Guangzhou, China). Total RNA was extracted using a Trizol reagent kit (Invitrogen, Carlsbad, CA, USA) according to the manufacturer’s protocol. The eukaryotic mRNA was enriched and reverse-transcribed into cDNA using random primers. Second-strand cDNA was synthesized by DNA polymerase. The cDNA libraries were amplified by PCR and sequenced using the Illumina NovaSeq 6000 platform.

For data analysis, raw sequencing reads were preprocessed using fastp (version 0.18.0) to eliminate adapter sequences and low-quality bases. Ribosomal RNA (rRNA) reads were excluded [41]. Cleaned reads were aligned to the reference genome, and gene expression levels were quantified using RSEM software (version 1.3.3). While transcript abundance was normalized as fragments per kilobase of transcript per million mapped reads (FPKM) for visualization, the raw read counts were used for differential expression analysis [42]. Differential expression analysis was performed using the DESeq2 R package (version 1.50.2), which utilizes a negative binomial distribution model for count-based statistics. Differentially expressed genes (DEGs) were identified based on thresholds of an absolute fold change ≥ 2 and a Benjamini–Hochberg adjusted *p*-value (FDR) < 0.05 to control for multiple testing error. Functional annotation of DEGs, including Gene Ontology (GO) and Kyoto Encyclopedia of Genes and Genomes (KEGG) pathway enrichment analyses, was performed using the clusterProfiler package (v4.8.3) [43,44].

### 2.20. Animal Experimental Design

Mice were treated following the experimental protocol approved by the Institutional Animal Care and Use at Guangdong University of Technology (SCXK/20211228). Six-week-old female C57BL/6 mice were obtained from the Guangdong Medical Laboratory Animal Center (Guangzhou, Guangdong, China, SCXK (Guangdong)/2023-0059). This specific strain and age were selected because C57BL/6 mice possess eumelanin-producing melanocytes similar to humans [45]. Furthermore, at six weeks of age, the dorsal hair follicles are synchronized in the telogen (resting) phase, ensuring that the skin background is unpigmented [46]. This allows for the precise evaluation of UVB-induced epidermal pigmentation without interference from follicular melanogenesis associated with the anagen (growth) phase.

Mice were maintained in a specific pathogen-free environment (23 ± 2 °C, 55% ± 10% humidity, 12-h light/dark cycle) with unrestricted access to standard diet and water. Before the experiment, animals were acclimatized for at least seven days. The dorsal skin of each mouse was shaved using Hair Removal Cream (Guangzhou Kerong Biological Co., Ltd., Guangzhou, China) to expose an area of approximately 2.5 × 3 cm (7.5 cm^2^), which was maintained hairless throughout the study.

Mice were randomly assigned to five groups (*n* = 6 per group). While doses were initially calculated based on body weight (mg/kg) for systemic safety consistency, they are reported here with corresponding concentrations and topical dosing densities (based on an average body weight of 20 g and 7.5 cm^2^ treated area) to ensure reproducibility:

1. Control Group: No UVB exposure; topically treated with 100 µL of PBS.
2. Model Group: UVB exposure; topically treated with 100 µL of PBS.
3. Positive Control: UVB exposure; topically treated with α-arbutin (Target: 3.3 mg/kg). This corresponds to a concentration of 0.66 mg/mL and a dosing density of 0.0088 mg/cm^2^.
4. SP-L Group: UVB exposure; topically treated with SP (Target: 1.67 mg/kg). This corresponds to a concentration of 0.33 mg/mL and a dosing density of 0.0044 mg/cm^2^.
5. SP-H Group: UVB exposure; topically treated with SP (Target: 3.3 mg/kg). This corresponds to a concentration of 0.66 mg/mL and a dosing density of 0.0088 mg/cm^2^.

All mice except the control group were exposed to UVB irradiation (0.15 J/cm^2^) three times a week (days 1, 3, and 5) for 3 weeks. The total cumulative UVB dose administered was approximately 1.35 J/cm^2^. Topical treatments (100 µL of PBS, α-arbutin, or SP solution) were administered daily to the shaved dorsal area for the entire 5-week experimental period. On days involving UVB exposure (weeks 1–3), the topical treatment was applied 30 min after irradiation; on non-irradiation days, it was applied at the same time of day. During the final two weeks (weeks 4–5), UVB irradiation was ceased, and mice continued to receive only the daily topical treatments to assess pigment recovery. After 5 weeks, all mice were sacrificed, and the dorsal skin samples were collected for analysis. Details of the animal experimental process are presented in Supplementary Figure S1.

### 2.21. Histopathological Analysis

The mice’s dorsal skin brightness (L* value) was measured using a skin colorimeter on the last day of each week, and body weights were recorded. After the mice were sacrificed, the dorsal skin tissues were excised using surgical scissors. Full-thickness skin specimens (containing both epidermis and dermis) were immediately fixed in 4% paraformaldehyde (PFA) containing 0.1 M sodium phosphate buffer for 24 h. The tissues were then dehydrated in graded ethanol, cleared in xylene, and embedded in paraffin wax according to standard protocols. Serial sections of 4 µm thickness were obtained using a microtome. The histological structure and skin architecture were analyzed using Hematoxylin and Eosin (H&E) staining [47]. To evaluate pigmentation levels, melanin granules were visualized using Fontana-Masson staining [38].

### 2.22. Inflammatory Hyperpigmentation-Related Indicators of Skin Tissue

The content of IL-18, IL-33, GM-CSF, and PGE-2 in the skin was determined using commercial kits and following the manufacturer’s protocols (Elabscience Biotechnology Co., Ltd., Wuhan, China). The absorbance of all samples was measured at 450 nm using a microplate reader.

### 2.23. Immunohistochemistry Analysis

Before dewaxing, the tissue chip was placed in a 70 °C constant temperature box overnight and immersed in dimethylbenzene and ethanol for dewaxing and hydration. Antigen repair was performed using high temperature and high pressure, maintaining it for 2 min, followed by washing with PBS five times. In each section, 50 μL of 3% hydrogen peroxide solution was added to block the endogenous peroxidase, and then the sections were incubated at room temperature for 10 min, followed by washing with PBS three times. Additionally, the sections were incubated overnight at 4 °C with rabbit primary antibodies raised against TYR (1:1000) and p-CREB (1:1000). The primary antibodies were labeled using the EnVision™ combination with the chromogenic agent DAB. All images were captured using an FV1000 laser confocal inverted microscope.

### 2.24. Statistical Analysis

All values obtained from at least three independent experiments are presented as the mean ± standard deviation (SD). Statistical analysis was conducted using one-way ANOVA followed by Tukey’s post hoc test, utilizing the Statistical Packages for the Social Sciences (SPSS) software (version 26.0, SPSS Inc., Chicago, IL, USA). The statistical significance of effects is indicated as follows: * *p* < 0.05, ** *p* < 0.01, and *** *p* < 0.001.

## 3. Results

### 3.1. Characterizations of SP

UHPLC and ESI-MS initially analyzed the chemical composition of SP. Figure 1 presents the total ion mass spectrum of the purified SP, revealing its molecular weight distribution. The spectrum indicates that the primary components of SP possess molecular weights predominantly below 1000 Da. This finding is significant because low-molecular-weight peptides have been reported to exhibit greater efficacy in inhibiting melanin production compared to larger peptides, an effect potentially attributed to the increased accessibility or exposure of their constituent active amino acid residues [48].

As shown in Table 1, we found that main peptide sequences comprising SP were Tyr-Leu-Lys (confidence: 88.3%), Gly-Leu-Tyr (confidence: 70.6%), Ile-Pro-Lys (confidence: 85.7%), Cys-Arg-Val-Gly-Ser-Thr (confidence: 53.6%), Val-Pro-Leu (confidence: 75.6%), Ala-Ile (confidence: 61.6%), Ala-Leu-Asp-Asp (confidence: 46.8%), Gly-Ala-Leu (confidence: 66.3%), Ala-Leu-Val-Arg-Phe-Tyr (confidence: 79.6%). The identified sequences were confirmed to match known *Spirulina* protein sequences documented in the UniProt Knowledgebase. (Swiss-Prot, TrEMBL, and PIR-PSD; http://www.uniprot.org/uniprot/K1RWN4 (accessed on 15 March 2025)). Schurink M et al. [49] demonstrated that short peptides containing L-Tyr at the C-terminus exhibit enhanced inhibitory activity against TYR. Consequently, the Gly-Leu-Tyr, identified in SP, suggests a contribution to TYR inhibition. Moreover, peptides exhibiting intense TYR inhibitory activity consistently contain one or more arginine (Arg) residues, typically associated with phenylalanine (Phe) or a combination of alanine (Ala) and leucine (Leu) [49]. In this study, the Ala-Leu-Val-Arg-Phe-Tyr and Ala-Leu-Asp-Asp, also identified in SP in this study, aligns well with these structural characteristics. The identification of peptides within SP exhibiting these specific structural features, known to be associated with TYR inhibition, supports the further study of their potential contribution in reducing melanin formation.

Downregulating melanin production using amino acids, peptides, and their analogs is a prominent research area [16]. The inhibitory effects of peptides on melanin production are closely associated with their amino acid composition and sequence. The amino acid composition analysis indicated that SP contains 17 amino acids (72.72 g/100 g), including many hydrophobic and TYR-inhibitory types linked to reduced melanin synthesis (Supplementary Table S1). Notably, it has all ten reported TYR-inhibiting amino acids, totaling 40.68 g/100 g (56.19% of total amino acids). This high proportion of anti-melanogenic amino acids supports SP’s potential to inhibit TYR activity and merits further study.

### 3.2. Enzyme Kinetics of Tyrosinase Inhibition Mechanism

#### 3.2.1. Tyrosinase-Inhibitory and Copper-Chelating Activities of SPs

Melanin synthesis is mainly regulated by tyrosinase (TYR), a rate-limiting enzyme possessing monophenolase and diphenolase activities [50]. In this study, the skin-whitening potential of SP was evaluated by examining its inhibitory effects on both activities. a-Arbutin, a clinically used whitening agent, and Tea polyphenols, a representative metal chelator, were employed as positive controls [51,52,53]. SP inhibited TYR monophenolase activity in a dose-dependent manner, with an IC50 value of 0.807 mg/mL (Supplementary Figure S2a,b), which was slightly weaker than that of a-Arbutin (IC_50_ = 0.516 mg/mL). In contrast, SP showed strong inhibition toward di-phenolase activity, exhibiting an IC_50_ of 0.328 mg/mL (Supplementary Figure S2c,d), comparable to and even exceeding that of a-Arbutin (IC_50_ = 0.335 mg/mL) at higher concentrations.

Considering that copper ions are essential cofactors for TYR catalysis, the cop-per-chelating ability of SP was further investigated. As shown in Supplementary Figure S2e,f, SP exhibited potent copper-chelating activity with an IC_50_ of 0.477 mg/mL, which was markedly stronger than that of Tea polyphenols (IC_50_ = 0.718 mg/mL). This result suggests that copper chelation may represent a key mechanism underlying the TYR inhibitory effect of SP, differing from the competitive inhibition mode of a-Arbutin. These findings are consistent with previous reports on bioactive peptides [34] and demonstrate that SP shows stronger inhibitory activity than rice bran peptides, while remaining less potent than jellyfish collagen peptides [54].

TYR is a copper-containing mixed-function oxidase with a hydrophobic cavity as its active center. The active center contains two Cu^2+^ ions, coordinated by three histidine each. An oxygen bridge connects the Cu^2+^ ions, and TYR’s binding activity depends on the oxidation states of copper. To further verify the inhibitory effect and mechanism of SP on TYR activity, we measured its ability to chelate copper ions. Supplementary Figure S2e,f illustrate the percentage of Cu^2+^ ion chelation rates and the IC_50_ values for the Cu^2+^ ion chelation capacity of SP at various concentrations. Optimal copper ion chelation (84.99%) by SP is achieved at a concentration of 2 mg/mL. SP demonstrated copper-chelating activity with an IC_50_ of 0.477 mg/mL. The activity of TYR is regulated by its Cu^2+^ chelating center. When Cu^2+^ is bound, oxygen transfer is inhibited, thereby preventing its catalytic function and suppressing melanin production [55]. These findings indicate that SP effectively chelates Cu^2+^, potentially allowing it to enter the hydrophobic cavity of TYR and bind to the substrate’s binding site. The interaction with Cu^2+^ reduces its catalytic activity and inhibits TYR function.

#### 3.2.2. Inhibition of TYR by SP and Kinetic Analysis

To further elucidate the inhibitory mechanism of SP on TYR, we investigated the impact of SP on enzyme activity using L-DOPA as a substrate at a fixed concentration and varying the amount of TYR. The resulting enzyme reaction rates were plotted TYR concentrations in Supplementary Figure S3a, yielding seven lines intersecting at the origin. As SP concentration increased, the slope of these lines progressively decreased, indicating that SP exerts a reversible inhibitory mode on TYR. These findings suggest that SP inhibits TYR activity by diminishing its ability directly to oxidize L-DOPA [33,56].

To determine the specific inhibitory mechanism, we performed enzyme kinetics studies. A Lineweaver-Burk double reciprocal plot (Supplementary Figure S3b) was generated by plotting the reciprocal of the substrate concentration against the reciprocal of the reaction velocity at different SP concentrations. The intersection of the lines in the second quadrant indicated a mixed-type inhibition pattern. This was further substantiated by the kinetic parameters (Supplementary Table S2): with increasing SP concentrations, the Vmax value decreased (from 0.0432 to 0.0162 ∆OD/min, as reflected by the increase in 1/V_m_ in Supplementary Figure S3c) and the K_m_ value slightly increased (from 0.0212 to 0.0280 mol/L, as reflected in Supplementary Figure S3d). These trends confirm that SP interferes with both substrate binding and catalytic turnover.

To quantify the affinity of SP for the enzyme, the inhibitory constants were determined using secondary plots. The inhibitory constant for the free enzyme (K_i_) was calculated to be 2.68 mg/mL, while the inhibitory constant for the enzyme–substrate complex (K_is_) was 3.05 mg/mL (Supplementary Table S2). Unlike typical competitive inhibitors, the values of K_i_ and K_is_ were close in magnitude, suggesting that SP binds to both the free enzyme and the enzyme–substrate complex with comparable affinity. Although the slightly lower K_i_ indicates a marginal preference for the free enzyme, the substantial binding to the enzyme–substrate complex (indicated by the relatively low K_is_) confirms that SP exhibits a robust mixed-type inhibition mechanism. This finding is consistent with previous research by Wijaya C et al. [57], who reported similar mixed-type inhibition characteristics.

### 3.3. Antioxidant Activity of SPs

#### 3.3.1. ABTS+ and DPPH Scavenging Activity

**Figure 2:**
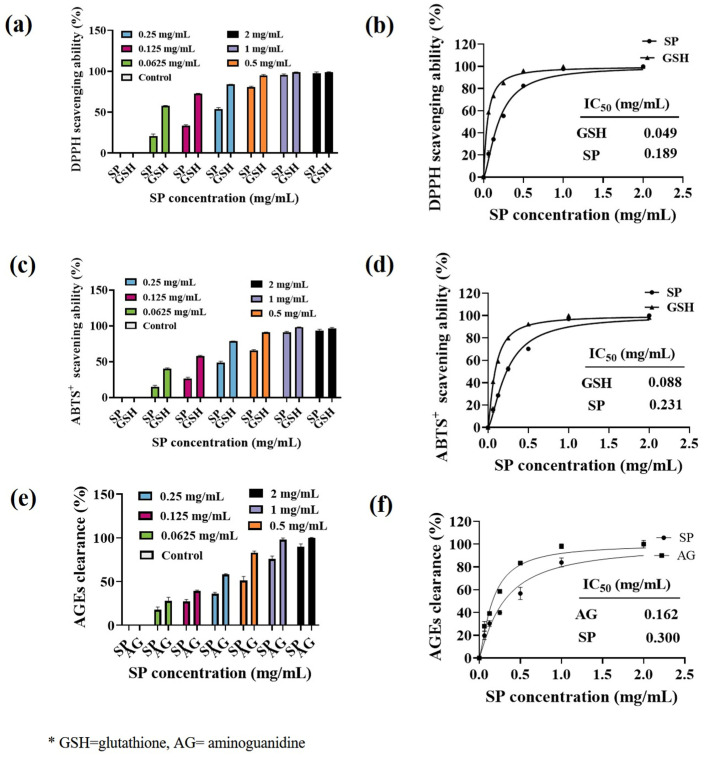
The scavenging ability of SP against 1,1-diphenyl-2-picrylhydrazyl radical (DPPH·) (**a**), 2,2′-azino-bis(3-ethylbenzothiazoline-6-sulfonate (ABTS+) (**c**), advanced glycation end products (AGEs) (**e**) at various concentrations along with their calculated IC_50_ values (**b**,**d**,**f**) SP from the fitting curves. * GSH = glutathione, AG = aminoguanidine.

Ultraviolet (UV) radiation induces the production of reactive oxygen species (ROS) in the skin, which is known to promote tyrosinase expression and subsequent melanin formation [58]. To investigate the antioxidant capacity of SP, we evaluated its ability to scavenge DPPH and ABTS free radicals. The DPPH radical scavenging activity of SP is shown in Figure 2a, and its corresponding IC_50_ value is presented in Figure 2b. Similarly, the ABTS radical scavenging activity is depicted in Figure 2c, with the IC_50_ value provided in Figure 2d. SP exhibited a concentration-dependent increase in both DPPH and ABTS radical scavenging abilities. For DPPH· scavenging ability (Figure 2a), SP demonstrated lower activity than the positive control glutathione (GSH) in the concentration range of 0–0.5 mg/mL. However, its scavenging ability gradually improved with increasing concentrations, reaching 80.55% at 0.5 mg/mL. Notably, SP demonstrates a higher DPPH· scavenging ability (IC_50_ = 0.189 mg/mL) compared to antioxidant peptides derived from Tilapia (*Oreochromis niloticus*) skin (IC_50_ = 2.56 mg/mL) [48].

As for the ABTS+· scavenging ability of SP (Figure 2c,d), the ABTS+· scavenging ability of SP (IC_50_ = 0.231 mg/mL) was weaker than that of positive control GSH (IC_50_ = 0.088 mg/mL). Nevertheless, at a concentration of 1 mg/mL, SP exhibited a substantial ABTS radical scavenging ability of 91.04%.

The antioxidant capacity of peptides is closely related to their structural characteristics, particularly molecular weight, amino acid composition, and sequence [59,60]. SP comprises short peptide molecules with 10 sequences of 2 to 6 amino acids and a molecular weight ranging from 200 to 800 Da, which likely contributes to its excellent antioxidant properties [61,62,63]. Previous research conducted by Wu et al. has indicated that Tyr and Gly at the C-terminal end of peptides enhance their antioxidant capabilities [64]. Including the sequence Gly-Leu-Tyr in SP may contribute to its antioxidant potential. Furthermore, specific combinations such as Arg with Phe or Ala with Leu, found in sequences like Ala-Leu-Val-Arg-Phe-Tyr and Ala-Leu-Asp-Asp, have also demonstrated significant antioxidant activity in SP [49]. These structural features of SP’s short peptide chains collectively enhance its antioxidant ability, which may contribute to the inhibition of melanin production in the skin.

#### 3.3.2. AGEs Scavenging Ability of SPs

Skin glycation, characterized by the accumulation of advanced glycation end products (AGEs), is exacerbated by UVB exposure and contributes to hyperpigmentation. UV irradiation of keratinocytes induces AGEs formation, which in turn stimulates melanin production through the ERK and CREB signaling pathways [65]. To evaluate the in vitro AGEs scavenging ability of SP, we established an MGO-glucose reaction system. As depicted in Figure 2e, the ability of SP to eliminate AGEs increased with increasing concentration. Curve fitting of the normalized data (Figure 2f) revealed an IC_50_ value of 0.300 mg/mL for SP in scavenging AGEs, while the positive control aminoguanidine (AG) exhibited an IC_50_ value of 0.162 mg/mL. Although SP showed lower AGEs scavenging activity compared to AG in the concentration range of 0–0.5 mg/mL, its capacity to scavenge AGEs reached over 80% at concentrations exceeding 1 mg/mL.

### 3.4. SP Modulation of TYR Activity and Melanogenesis in B16F10 Cells

#### 3.4.1. SPs Inhibit TYR Activity and Melanin Synthesis in B16F10 Cells

**Figure 3:**
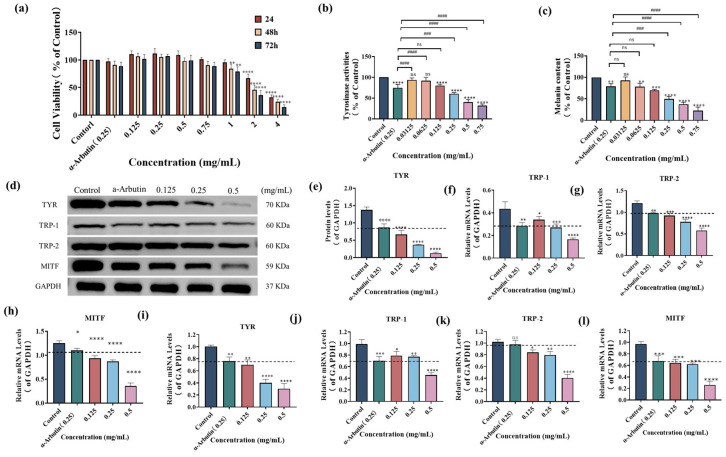
Effect of SP on the cell viability (**a**), tyrosinase (TYR) activities (**b**), and melanin contents (**c**) in mouse melanoma B16F10 cells. The effects of SP on enzymatic proteins and mRNA expressions in B16F10 cells (**d**–**f**). (**d**) The expressions of melanogenic enzymes and microphthalmia-associated transcription factor (MITF) were determined using Western blotting analysis in B16F10 cells. All bands were detected using ChemiDoc™ XRS + System. The impact of SP on the protein expression of TYR (**e**), tyrosinase-related protein 1 (TRP-1) (**f**), tyrosinase-related protein 2 (TRP-2) (**g**), and MITF (**h**). The impact of SP on the mRNA expression of TYR (**i**), TRP-1 (**j**), TRP-2 (**k**), and MITF (**l**) in B16F10 cells. Statistical results are expressed as the means ± SD of three independent experiments. *, **, *** and **** indicate *p* < 0.05, *p* < 0.01, *p* < 0.001 and *p* < 0.0001, respectively, compared with the control; ### and #### indicate *p* < 0.001 and *p* < 0.0001, compared with α-Arbutin group; ns indicates no significant difference. Dash lines are presented as α-Arbutin for reference.

To determine the appropriate concentration range for studying the effects of SP on melanogenesis, we first assessed its cytotoxicity in B16F10 cells using the MTT assay. As shown in Figure 3a, cell viability decreased significantly after exposure to 2–4 mg/mL SP for 24–72 h (*p* < 0.0001) or 1 mg/mL for 48–72 h (*p* < 0.01) when compared with those of the control group (*p* < 0.0001), indicating cellular toxicity at these concentrations. Based on these results, we selected a non-toxic concentration range of 0.03125–0.75 mg/mL for the subsequent experiments, as it did not significantly promote cell proliferation or induce significant toxicity.

Within this non-toxic concentration range, we investigated the impact of SP on intracellular TYR activity and melanin production in B16F10 cells (Figure 3b,c). As the concentration of SP increased from 0.03125 mg/mL to 0.75 mg/mL, there was a gradual decrease in intracellular TYR activity. At a concentration of 0.125 mg/mL, the remaining intracellular TYR activity was 80.01 ± 3.62% of the control group, which did not show a statistically significant difference compared to the positive drug α-arbutin (0.25 mg/mL). However, at a 0.25 mg/mL concentration, the remaining TYR activity was 60.33 ± 3.21%, significantly lower than the positive drug α-arbutin (0.25 mg/mL).

To further investigate the inhibitory effect of SP on melanin production, the relative content of residual melanin in B16F10 cells was determined at different concentrations of SP, as shown in Figure 3c. Melanin production was significantly suppressed by SP at a concentration of 0.0625 mg/mL, with an intracellular residual melanin content of 78.36%, comparable to α-arbutin (0.25 mg/mL). At 0.25 mg/mL, SP further reduced intracellular residual melanin content to 49.51%, demonstrating a more potent inhibitory effect than the positive drug α-arbutin. The most substantial inhibition of TYR activity (31.95%) and remaining melanin content (22.55%) was observed at a concentration of 0.75 mg/mL. These results suggest that SP effectively reduces melanin synthesis by inhibiting intracellular TYR activity in B16F10.

#### 3.4.2. SP Downregulates Expression of TYR-Related Proteins and mRNA in B16F10 Cells

Melanin biosynthesis is primarily regulated by three enzymes, namely TYR, tyrosine-related protein 1 (TRP-1), and tyrosine-related protein 2 (TRP-2) [58,66], with microphthalmia-associated transcription factor (MITF) playing a crucial role as transcriptional regulator of these enzymes [67,68,69]. To further elucidate the mechanism of SP’s inhibitory effect on melanogenesis, we investigated the inhibitory effects of SP on the expression levels of these melanin-related proteins and corresponding mRNA in B16F10 cells.

Western blot analysis (Figure 3d) revealed that SP down-regulated the protein expression levels of TYR, TRP-1, TRP-2, and MITF in a dose-dependent manner, as quantified by densitometry relative to GAPDH (Figure 3e–h). At 0.125 mg/mL, SP exhibited a more potent inhibitory effect on the expression of TYR, TRP-2, and MITF proteins compared to α-arbutin (0.25 mg/mL). At 0.25 mg/mL, SP’s inhibitory effect on TRP-1 protein expression is comparable to that of α-arbutin at the same concentration.

To further validate the inhibitory effect of SP on the transcriptional level, we performed Q-PCR to measure the mRNA expression levels of TYR, TRP-1, TRP-2, and MITF (Figure 3i–l). Consistent with the protein expression data, SP downregulated mRNA expression of these genes in a dose-dependent manner. At 0.125 mg/mL, SP exhibited more potent inhibitory effects on the mRNA expression of TYR and TRP-2 than α-arbutin (0.25 mg/mL). Furthermore, at a concentration of 0.25 mg/mL, SP’s inhibitory effect on MITF mRNA expression was comparable to that of α-arbutin at an equivalent concentration and slightly surpassed α-arbutin (0.25 mg/mL) in suppressing TRP-1 mRNA expression.

The results collectively indicate that SP inhibits melanin production in B16F10 cells by downregulating both the protein and mRNA transcription levels of key enzymes (TYR, TRP-1, TRP-2) and their major transcriptional regulator (MITF). This suggests that SP’s mechanism of action involves suppressing the transcription of these essential genes involved in melanin synthesis, thereby reducing the availability of these proteins and consequently inhibiting melanogenesis [70].

### 3.5. SP Modulates Melanogenesis-Related Signaling Pathways

#### 3.5.1. SP Downregulates cAMP-CREB Signal Pathway

**Figure 4:**
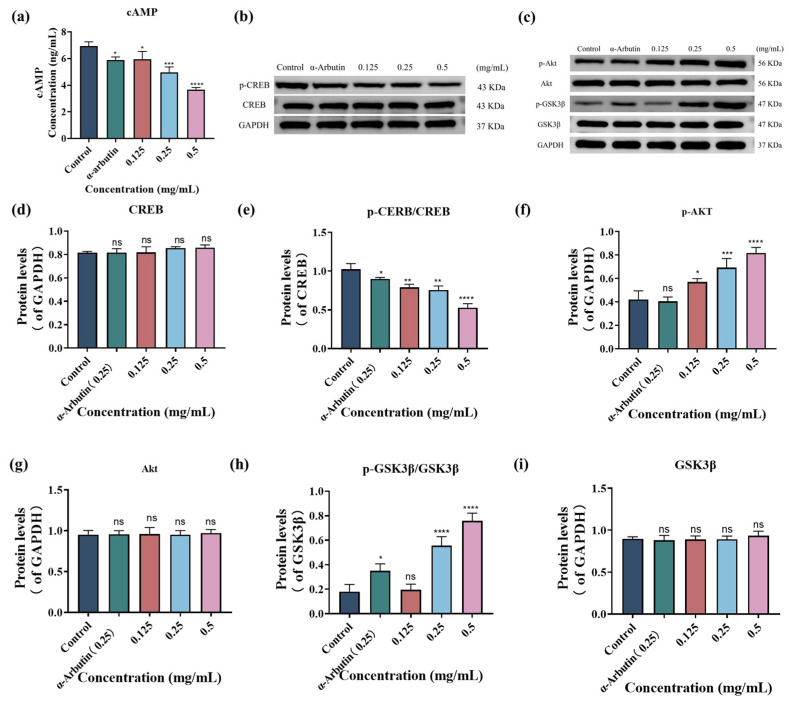
(**a**) SP dose-dependently downregulated cyclic adenosine monophosphate (cAMP) in B16F10 cells. Effects of different concentrations of SP on the protein expression of cAMP, cAMP response element-binding protein (CREB), protein kinase B (Akt) and glycogen synthase kinase-3 beta (GSK3β) signaling pathway in B16F10 cells (**b**–**g**). Densitometry graphs of the Western blot analyses for cAMP-CREB (**b**) and Akt/GSK3β (**c**) signaling pathway. Protein levels of p-CREB (**d**) and CREB (**e**), p-Akt (**f**), Akt (**h**), p-GSK3β/GSK3β (**i**), and GSK3β (**g**) were examined by Western blot. Statistical results are expressed as the means ± SD of three independent experiments. *, **, *** and **** indicate *p* < 0.05, *p* < 0.01, *p* < 0.001 and *p* < 0.0001, respectively, compared with the control group; ns indicates no significant difference.

To elucidate the mechanism by which SP inhibits melanin production in B16F10 cells, we first examined its effect on intracellular cyclic cAMP levels using ELISA. cAMP is a well-established intracellular second messenger that plays a crucial role in melanogenesis cascade through signal transduction [71]. In this study, cAMP was investigated as a key component of the cAMP-CREB signaling pathway. As shown in Figure 4a, AMP content decreased with increasing SP concentrations. Specifically, compared to the control group, treatment with SP at 0.125, 0.25, and 0.5 mg/mL resulted in cAMP levels of 5.96, 4.96, and 3.66 ng/mL, respectively. α-Arbutin (0.25 mg/mL), used as a positive control, reduced cAMP levels to 5.87 ng/mL, indicating that SP exhibited a more substantial inhibitory effect on cAMP expression than α-arbutin at comparable concentrations.

One of the key downstream targets of cAMP signaling is the transcription factor CREB (cAMP response element-binding protein). Protein kinase A (PKA), activated by cAMP, phosphorylates CREB, which in turn activates MITF [71,72]. To further investigate SP’s mechanism, we evaluated its impact on intracellular CREB phosphorylation using Western blot analysis (Figure 4b,d,e). The results revealed that SP inhibited CREB phosphorylation in a dose-dependent manner. These results suggest that SP downregulates TYR expression by modulating the cAMP-CREB signaling pathway, inhibiting melanin production.

#### 3.5.2. SP Activates Akt/GSK3β Signal Pathway

To further investigate the inhibitory mechanisms of SP on melanin production, we examine its effects on the Akt/GSK3β signaling pathway. While research on the signaling pathways of TYR-inhibiting peptides has often focused on cAMP-CREB signaling [73,74,75], the Akt/GSK3β pathway is also recognized as a critical regulator of MITF transcriptional activity. Activation of the Akt/GSK3β pathway has been shown to suppress melanin accumulation in melanocytes [76]. In our study, Western blot analysis (Figure 4c) demonstrated a significant increase in the phosphorylation levels of Akt (p-Akt, Figure 4f,g) and GSK-3β (p-GSK-3β, Figure 4h,i) in SP-treated cells compared to the control group. These results indicate that SP activates the Akt/GSK3β signaling pathway, leading to the phosphorylation of GSK-3β. Phosphorylation of GSK-3β typically results in its inactivation, which in turn prevents the phosphorylation of MITF, ultimately leading to MITF degradation and inhibition of melanin production [77].

### 3.6. Transcriptional Analysis by RNA Sequencing

#### 3.6.1. Analysis of Differentially Expressed Genes (DEGs)

**Figure 5:**
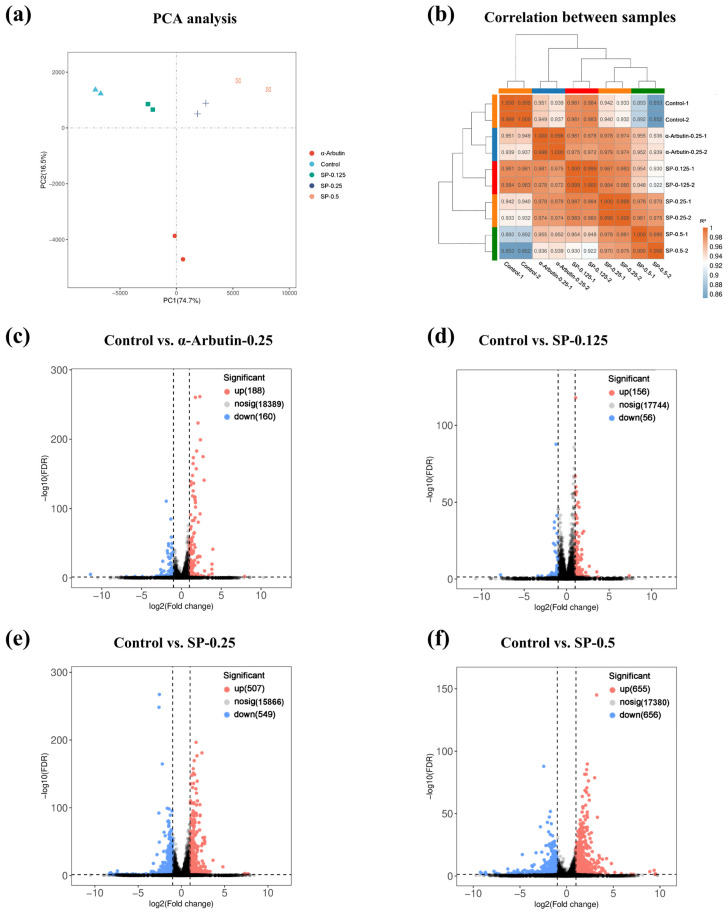
Quality assessment and differential expression analysis of transcriptomic data in B16F10 cells. (**a**) principal component analysis (PCA) plot showing distinct clustering of Control, α-arbutin (0.25 mg/mL), and SP-treated groups (0.125–0.5 mg/mL), with biological replicates tightly clustered (*n* = 3). (**b**) Heatmap of Pearson correlation coefficients (r > 0.95) confirming high reproducibility across replicates. (**c**–**f**) Volcano plots of DEGs (|log_2_FC| > 1, FDR < 0.05) in SP-treated groups versus Control. Upregulated (red) and downregulated (blue) genes are highlighted. Key pathways (e.g., melanogenesis, oxidative stress) were enriched in SP-responsive DEGs.

To elucidate the molecular mechanisms underlying the inhibitory effect of SP on melanogenesis, transcriptome sequencing was performed on B16F10 cells treated with vehicle control, α-arbutin (positive control, 0.25 mg/mL), or varying concentrations of SP (0.125, 0.25 and 0.5 mg/mL). Initial quality assessment of the sequencing data was conducted using Principal Component Analysis (PCA) and Pearson correlation analysis. PCA revealed distinct clustering patterns, with a clear separation between the different treatment groups (control, α-arbutin, SP-0.125, SP-0.25, and SP-0.5) in the principal component space, indicating unique transcriptomic profiles associated with each treatment (Figure 5a). Furthermore, samples within each experimental group clustered tightly together, demonstrating low intra-group variability (Figure 5a). Complementary analysis using Pearson correlation coefficients confirmed high biological reproducibility, showing strong correlations (R^2^ ≈ 1) among replicates within the same group, contrasted with lower correlations between different groups (Figure 5b). Collectively, these analyses validate the quality and reliability of the transcriptome dataset and confirm that SP treatment induces significant and distinct alterations in gene expression patterns in B16F10 cells compared to both control and α-arbutin treated cells.

Differential gene expression (DEG) analysis compared each treatment group to the vehicle control to identify genes transcriptionally regulated by SP treatment. Volcano plots were generated to visualize the magnitude (log2 Fold Change; *x*-axis) and statistical significance (-log10 FDR; *y*-axis) of expression changes for all detected genes (Figure 5c–f). Using stringent criteria (|log2FC| > 1 and FDR < 0.05), genes were classified as significantly upregulated (red dots) or downregulated (blue dots).

Compared to control cells, α-arbutin (0.25 mg/mL) treatment resulted in 188 upregulated and 160 downregulated DEGs (Figure 5c). Treatment with SP induced a dose-dependent increase in the number of DEGs: SP at 0.125 mg/mL yielded 156 upregulated and 56 downregulated DEGs; SP at 0.25 mg/mL resulted in 507 upregulated and 549 downregulated DEGs; and SP at 0.5 mg/mL led to 655 upregulated and 656 downregulated DEGs (Figure 5d–f). The substantial number of DEGs observed, particularly at the highest SP concentration, underscores the significant impact of SP on the B16F10 cell transcriptome.

Furthermore, hierarchical clustering analysis revealed distinct expression patterns across treatment groups. Notably, the expression levels of key melanogenesis-related genes, *Mitf* and *Tyr*, were markedly downregulated in all SP-treated groups relative to the control. This downregulation exhibited clear dose-dependency, becoming more pronounced with increasing SP concentrations (visualized as blue regions in the heatmap, Supplementary Figure S4), strongly suggesting a concentration-dependent inhibitory effect of SP on essential melanogenic pathways.

#### 3.6.2. Enrichment Analysis of GO Function and KEGG Pathway

**Figure 6:**
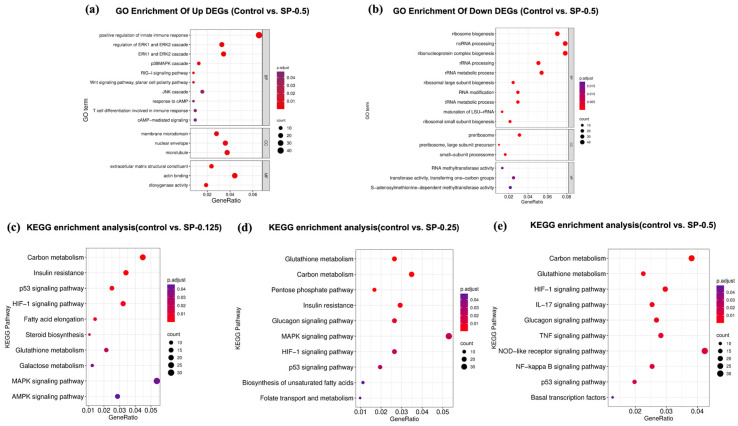
Identification of key biological functions and pathways associated with SP treatment. gene ontology (GO) analysis highlights significantly enriched functional terms (FDR < 0.05) associated with (**a**) upregulated and (**b**) downregulated DEGs following SP (0.5 mg/mL) treatment relative to Control. Kyoto Encyclopedia of Genes and Genomes (KEGG) pathway analysis reveals significantly enriched pathways (FDR < 0.05) associated with DEGs from comparisons of (**c**) SP (0.125 mg/mL), (**d**) SP (0.25 mg/mL), and (**e**) SP (0.5 mg/mL) treatments versus Control, illustrating potential dose-dependent effects.

To explore the functional implications of the observed transcriptional changes, Gene Ontology (GO) enrichment analysis was performed on the DEGs identified in the SP-0.5 group relative to the control. For the upregulated DEGs, significant enrichment was observed across the three main GO domains (Figure 6). Key enriched terms included “membrane microdomain” (Cellular Component), “extracellular region” (Molecular Function), and biological processes such as “ERK1 and ERK2 cascade”, “p38 MAPK cascade”, “response to cAMP”, and “cAMP-mediated signaling” (Biological Process). The enrichment in MAPK cascade terms is notable, given that p38 MAPK activation can phosphorylate MITF and enhance *Tyr* transcription. Similarly, the enrichment related to cAMP signaling aligns with the established role of the CREB-Tyr axis in melanogenesis.

To elucidate potential dose-dependent mechanisms, KEGG pathway enrichment analysis was conducted on DEGs from each SP concentration group (0.125, 0.25, and 0.5 mg/mL) compared to the control (Figure 6). While the top enriched pathways across all groups involved functions like inflammation, metabolism, and immune regulation, distinct patterns emerged. DEGs from lower SP concentrations (0.125, 0.25 mg/mL) were significantly enriched in the MAPK signaling and p53 signaling pathways. In contrast, DEGs identified at the highest concentration (SP-0.5 mg/mL) showed significant enrichment primarily in the TNF and NF-κB signaling pathways. These findings suggest a concentration-dependent shift in SP’s mode of action: lower doses may influence cellular homeostasis potentially via pathways involving p53 signaling, while the higher dose appears to predominantly modulate inflammatory networks (TNF, NF-κB). This high-dose effect could potentially involve modulation of the JNK/p38 cascade, thereby impacting the expression of *Mitf* and its key downstream target *Tyr*, suggesting multi-pathway intervention in melanocyte regulation.

Finally, to investigate regulatory relationships within key melanogenesis networks, we constructed a co-expression network. This analysis focused on DEGs (|log2FC| ≥ 1, FDR < 0.05) from the SP-0.5 group implicated in MAPK, JAK-STAT, PI3K/AKT, and cAMP signaling pathways. Significant pairwise correlations (Pearson |r| > 0.6, FDR-adjusted *p* < 0.05) were calculated across samples and visualized as a correlation matrix heatmap (Supplementary Figure S5), where dark red and dark blue indicate strong positive and negative correlations, respectively. This network analysis revealed a significant positive correlation between the key melanogenic enzyme TYR and CREB3, a transcription factor within the cAMP pathway. Conversely, Tyr expression was significantly negatively correlated with FOXO3, a downstream effector of the PI3K/AKT pathway. This negative correlation aligns with known biology, where PI3K/AKT signaling can inhibit FOXO3 nuclear translocation, potentially relieving its transcriptional repression of target genes like *Tyr*.

### 3.7. SPs Protect UVB-Induced Injury in C57BL/6 Mice

#### 3.7.1. Body Weight of C57BL/6 Mice Treated with SPs

**Figure 7:**
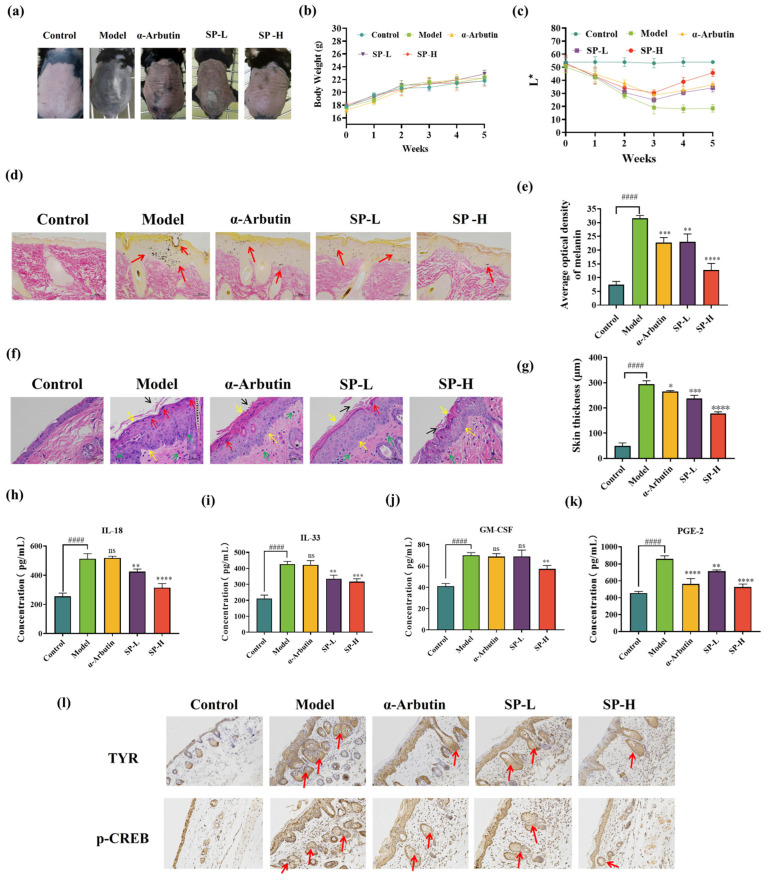
Effects of SP on skin morphological changes (**a**), body weight (**b**), and skin brightness (**c**) in the dorsal skin of UVB-irradiated C57BL/6 mice. Fontana-Masson staining of UVB-irradiation-induced hyperpigmentation in C57BL/6 mice skin. Melanin pigments (as indicated by the arrows) were stained black (**d**) (scale bar = 50 μm) and semi-quantification (**e**). Hematoxylin and Eosin (H&E) staining of UVB-irradiation-induced histopathological changes in C57BL/6 mouse skin, tissue morphology (**f**) (scale bar = 50 μm) and semi-quantification (**g**). The effect of SP on pro-inflammatory cytokines (**h**) interleukin 33 (IL-33), (**i**) interleukin 18 (IL-18), (**j**) granulocyte-macrophage colony-stimulating factor (GM-CSF), and (**k**) prostaglandin E2 (PGE-2) contents. The effects of SP on UVB-induced epidermal TYR and p-CREB expressions in the dorsal skin of C57BL/6 mice, malanin illustrated with arrows. (**l**). Representative photographs of epidermal TYR and epidermal p-CREB distribution by IHC staining (400×). Data was expressed as mean ± SD (*n* = 6). *, **, *** and **** indicate *p* < 0.05, *p* < 0.01, *p* < 0.001 and *p* < 0.0001, respectively, compared with the model group. #### indicate *p* < 0.0001, compared with the control group; ns indicates no significant difference.

To evaluate the potential impact of SP on healthy mice throughout the UVB-induced skin pigmentation therapy, the body weight of the mice was measured after each weekly treatment, as seen in Figure 7b. Throughout the 5-week experimental period, the control group exhibited a consistent growth trend. In contrast, there were no statistically significant differences in body weight among the model group, positive group (α-arbutin), SP-L group, and SP-H group compared to the control group. It indicates no significant impact on mouse body weight under an irradiation dose of 150 mJ/cm^2^ with treatments involving α-arbutin and SP.

#### 3.7.2. Morphology and Pigmentation in C57BL/6 Mouse Treated with SP

Gross morphological examination of the dorsal skin upon dissection revealed distinct differences between the experimental groups (Figure 7a). Compared to the untreated control group, the skin of mice exposed solely to UVB irradiation (model group) exhibited pronounced hyperpigmentation, apparent thickening, and increased keratinization. Treatment with SP mitigated these UVB-induced alterations, resulting in a visible reduction in melanin accumulation in the dorsal skin. Notably, high-dose SP (SP-H) significantly attenuated UVB-induced pigment deposition, displaying qualitatively superior efficacy compared to the positive control group treated with α-arbutin.

To quantitatively evaluate skin pigmentation, L* values, which represent skin lightness (where higher values indicate lighter skin and lower values indicate darker skin), were measured in the treated dorsal skin areas using a skin colorimeter [78,79]. The results are presented in Figure 7c. Over the 5-week study period, the model group showed a significant decrease in skin L* values relative to the control group, confirming substantial melanogenesis and skin darkening induced by UVB exposure. Conversely, SP administration led to a marked increase in L* values, indicating an attenuation of UVB-induced pigmentation. Comparative analysis showed that the effect of low-dose SP (SP-L) on skin lightness was not significantly different from that of the positive control (1% α-arbutin). However, SP-H treatment resulted in significantly higher L* values compared to α-arbutin, indicating superior skin-lightening efficacy.

Collectively, these morphological observations and quantitative colorimetric measurements demonstrate that SP treatment effectively suppresses UVB-induced hyperpigmentation in the dorsal skin of C57BL/6 mice.

#### 3.7.3. Histological Assessment of Melanin Content via Fontana-Masson Staining

To further evaluate the effect of SP on melanin production and distribution within the skin tissue, dorsal skin samples collected at the study endpoint were subjected to Fontana-Masson staining. As illustrated in Figure 7d, control group skin sections displayed minimal melanin staining. In contrast, the epidermis of mice in the UVB-exposed model group showed abundant, large, and densely packed melanin granules. These granules were distributed throughout the epidermal layers (stratum corneum, granular layer, and spinous layer), with apparent accumulation in the uppermost stratum corneum, suggesting upward migration.

Compared to the model group, SP treatment led to a marked reduction in both the quantity and density of melanin granules in the dorsal epidermis. Notably, the melanin-reducing effect of SP-L was comparable to that of α-arbutin, with both treatments showing significantly less melanin deposition than the model group. Furthermore, SP-H treatment resulted in a pronounced inhibition of melanin production.

To quantify these observations, the average optical density (AOD) of melanin staining within the sections was analyzed using Image-Pro software (version 1.53k; National Institutes of Health, Bethesda, MD, USA). This quantitative analysis, presumably represented alongside the micrographs in Figure 7d, revealed that SP-H treatment reduced melanin AOD by approximately 40% compared to the model group. Therefore, these histological and quantitative findings confirm that SP effectively inhibits UVB-induced melanin deposition in the skin.

#### 3.7.4. SP Mitigates UVB-Induced Skin Inflammation

Skin inflammation, often linked to pigmentation changes [80,81], is a response to stimuli like UVB irradiation [82,83,84]. We examined SP’s anti-inflammatory effects using hematoxylin–eosin (HE) staining (Figure 7f). Compared to controls, UVB exposure (model group) induced pronounced hyperkeratosis, epidermal thickening (quantified in Figure 7g, acanthosis (thickening of granular/spinous layers), and significant inflammatory cell infiltration, including abscess formation (Figure 7f, arrows). SP treatment markedly reduced these inflammatory signs. Notably, SP-L demonstrated anti-inflammatory efficacy superior to the positive control, α-arbutin.

These histological findings (Figure 7f), alongside the melanin reduction data (Figure 7d) and skin lightening results (Figure 7c,e), suggest SP inhibits both melanogenesis and inflammation. Since post-inflammatory hyperpigmentation (PIH) links these processes [85,86], we investigated the underlying mechanism by measuring key inflammatory mediators (IL-18, IL-33, PGE-2, GM-CSF) in skin tissue via ELISA (Figure 7h–k). UVB irradiation significantly elevated all four mediators compared to controls. SP treatment caused a dose-dependent reduction in these cytokines, with SP-H showing significantly greater inhibition of IL-18, IL-33, and GM-CSF than α-arbutin.

Keratinocytes release these factors (IL-18, IL-33, PGE-2, GM-CSF) upon UVB exposure [87]. IL-18, IL-33, and PGE-2 are known to promote melanogenesis, primarily by activating the cAMP/PKA/CREB pathway, ultimately upregulating MITF and tyrosinase-related proteins (TYRPs) [88,89,90]. GM-CSF can stimulate melanocyte proliferation and melanin synthesis via MAPK pathways [90]. Our results show that SP significantly suppressed the UVB-induced elevation of IL-18, IL-33, PGE-2, and GM-CSF. We propose that by reducing these pro-inflammatory signals, SP attenuates downstream activation of both the cAMP/PKA/CREB and MAPK pathways. This likely leads to decreased expression of MITF and melanogenesis enzymes (e.g., TYR, TRP-1, TRP-2), consequently inhibiting melanin production.

#### 3.7.5. SP Reduces TYR and p-CREB Protein Expression in UVB-Irradiated Mouse Skin

To further elucidate the molecular mechanism underlying SP’s anti-pigmentation effect, the expression levels of TYR, a key melanogenic enzyme, and phosphorylated CREB (p-CREB), an indicator of PKA pathway activation, were examined in dorsal skin tissues via immunohistochemistry (IHC) (Figure 7l). Consistent with UVB’s known effects, irradiated skin (model group) exhibited markedly increased epidermal staining for both p-CREB and TYR compared to the control group. Topical SP administration dose-dependently suppressed this UVB-induced upregulation, resulting in visibly reduced levels of both p-CREB and TYR expression.

These IHC findings corroborate the proposed link between inflammation, signaling pathways, and pigmentation. As demonstrated previously, SP treatment significantly lowers the levels of UVB-induced pro-inflammatory mediators (e.g., IL-18, IL-33, PGE-2) known to activate the PKA-CREB pathway [88,89,90]. The observed reduction in p-CREB levels (Figure 7l) provides direct in vivo evidence that SP dampens this signaling cascade. Consequently, the reduced p-CREB activation leads to lower expression of its downstream target TYR (Figure 7l), thus inhibiting melanin synthesis. This suggests that SP alleviates UVB-induced hyperpigmentation, at least in part, by mitigating the inflammation–PKA-CREB-TYR axis. Notably, although many low-molecular-weight tyrosinase inhibitors can be more potent in isolated enzyme assays [51,91], our in vivo evidence supports that SP provides a broader anti-hyperpigmentation profile by concurrently suppressing inflammatory cues and the downstream PKA–CREB–TYR signaling axis, which may confer practical advantages for topical application. Nonetheless, dedicated in vitro validation under UVB irradiation would further strengthen the mechanistic link observed in vivo. Future studies should validate SP in UVB-irradiated melanocytes and keratinocyte–melanocyte models to better mimic the UV-driven microenvironment and further substantiate the inflammation–PKA/CREB–TYR axis in vitro.

## 4. Conclusions

**Figure 8:**
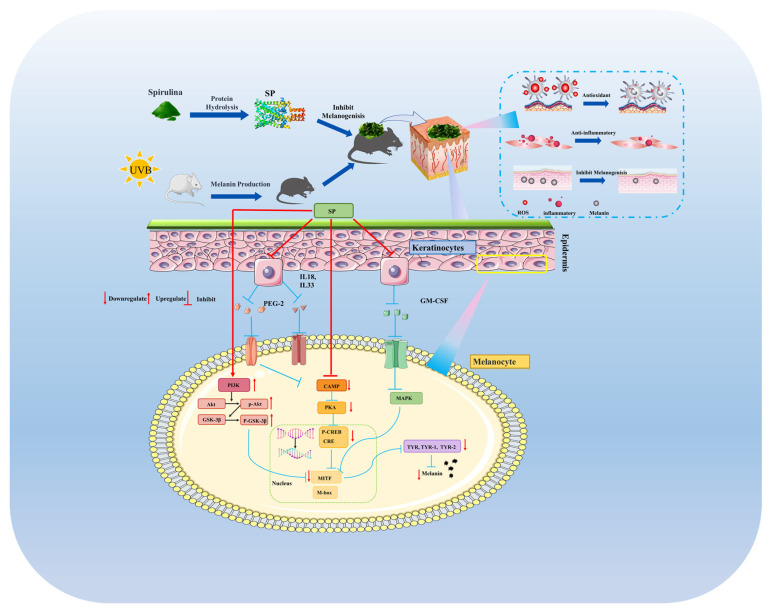
SP inhibited UVB-induced melanogenesis in mice via downregulating cAMP/PKA/CREB cascade and activating PI3K/Akt/GSK-3β pathway.

This investigation establishes SP, a newly characterized peptide composition, as a potent agent against hyperpigmentation, distinguished by its comprehensive, multi-target mechanism of action. Beyond direct, mixed-type inhibition of tyrosinase, SP uniquely demonstrates the ability to suppress melanin synthesis by concurrently modulating two critical intracellular signaling cascades—the cAMP/PKA/CREB and PI3K/Akt/GSK-3β pathways—leading to diminished expression of key melanogenic regulators like MITF and TYR within melanocytes (Figure 8). Crucially, these in vitro findings were validated in vivo, where SP not only reduced UVB-induced hyperpigmentation but also significantly ameliorated associated skin inflammation. A key insight from this work is the demonstration that SP’s anti-inflammatory efficacy, marked by the suppression of pro-inflammatory mediators, directly translates to reduced melanogenesis in vivo via downregulation of the CREB phosphorylation and subsequent TYR expression. This demonstrated the interplay between anti-inflammatory and anti-melanogenic actions, highlighting SP’s significant therapeutic potential, particularly for complex pigmentation disorders like post-inflammatory hyperpigmentation where controlling inflammation is paramount. By elucidating this integrated mechanism, our study positions SP as a highly promising candidate for further development in dermatological applications targeting aberrant pigmentation.

Although SP showed multi-target anti-melanogenic and anti-inflammatory activity, the specific bioactive peptides driving these effects were not identified. Pathway-level causality for cAMP/PKA/CREB and PI3K/Akt/GSK-3β modulation remains to be confirmed using targeted intervention (e.g., selective inhibitors, genetic knockdown/overexpression, or reporter assays). While in vivo evidence was obtained from a UVB-induced mouse model with limited follow-up, longer-term efficacy, relapse after withdrawal, and comprehensive dermal safety are needed. Future studies should validate SP in UVB-irradiated melanocytes and keratinocyte–melanocyte models to better mimic the UV-driven microenvironment and substantiate the inflammation–PKA/CREB–TYR axis in vitro, and further confirm antioxidant-linked mechanisms in human-relevant skin models while optimizing topical formulation, stability, and skin penetration.

## Figures and Tables

**Table 1:** Main peptide sequence profiles of SPs.

Peptide Sequence	Formula	Molecular Weight (Da)	Retention Time (min)	Confidence	Content
Tyr-Leu-Lys	C_21_H_34_N_4_O_5_	422.2529	7.383	88.3%	6.86%
Gly-Leu-Tyr	C_17_H_25_N_3_O_5_	351.1794	8.737	70.6%	3.73%
Ile-Pro-Lys	C_17_H_32_N_4_O_4_	356.2424	1.261	85.7%	3.67%
Cys-Arg-Val-Gly-Ser-Thr	C_23_H_43_N_9_O_9_S	621.7074	4.336	53.6%	3.86%
Val-Pro-Leu	C_16_H_29_N_3_O_4_	327.2158	9.753	75.6%	2.66%
Ala-Ile	C_9_H_18_N_2_O_3_	202.1317	3.411	61.6%	2.04%
Ala-Leu-Asp-Asp	C_17_H_28_N_4_O_9_	432.1856	3.789	46.8%	1.78%
Gly-Ala-Leu	C_11_H_21_N_3_O_4_	259.3021	1.066	66.3%	1.71%
Ala-Leu-Val-Arg-Phe-Tyr	C_38_H_57_N_9_O_8_	767.9147	6.886	79.6%	1.66%

## Data Availability

The original contributions presented in this study are included in the article/Supplementary Material. Further inquiries can be directed to the corresponding authors.

## Supplementary Materials

The following supporting information can be downloaded at: https://www.mdpi.com/article/10.3390/antiox15020181/s1, Figure S1: Process of the animal experiment.; Table S1: The composition of hydrolyzed amino acids in SP; Figure S2: Inhibition of SP against tyrosinase; Figure S3: Inhibitory mechanism of SP on TYR; Table S2: Kinetic parameters of TYR inhibition; Figure S4: Distinct gene expression profiles revealed by heatmap analysis; Figure S5: Correlation matrix heatmap visualizing co-expression patterns among melanogenesis-related DEGs.

## Abbreviations

The following abbreviations are used in this manuscript:

ABTS	2,2′-azino-bis(3-ethylbenzothiazoline-6-sulfonate)
AGE	advanced glycation end product
AGEs	advanced glycation end products
AG	aminoguanidine
Akt	protein kinase B
BSA	bovine serum albumin
cAMP	cyclic adenosine monophosphate
CREB	cAMP response element-binding protein
Cu^2+^	copper ion
DEGs	differentially expressed genes
DMEM	Dulbecco’s modified Eagle’s medium
DMSO	dimethyl sulfoxide
DPPH	1,1-diphenyl-2-picrylhydrazyl
EGFR	epidermal growth factor receptor
ERK	extracellular signal-regulated kinase
FBS	fetal bovine serum
FDR	false discovery rate
FPKM	fragments per kilobase of transcript per million mapped reads
GAPDH	glyceraldehyde 3-phosphate dehydrogenase
GM-CSF	granulocyte-macrophage colony-stimulating factor
GO	gene ontology
GSH	glutathione
GSK-3β	glycogen synthase kinase-3 beta
IC50	half-maximal inhibitory concentration
IL-18	interleukin 18
IL-33	interleukin 33
JAK-STAT	Janus kinase-signal transducer and activator of transcription
KEGG	Kyoto Encyclopedia of Genes and Genomes
MAPK	mitogen-activated protein kinase
MC1R	melanocortin 1 receptor
MGO	methylglyoxal
MITF	microphthalmia-associated transcription factor
MMP	matrix metalloproteinase
mRNA	messenger RNA
MSH	melanocyte-stimulating hormone
MTT	3-(4,5-dimethylthiazol-2-yl)-2,5-diphenyl tetrazolium bromide
NF-κB	nuclear factor kappa-light-chain-enhancer of activated B cells
PBS	phosphate-buffered saline
PCA	principal component analysis
PGE-2	prostaglandin E2
PI3K	phosphatidylinositol 3-kinase
PKA	protein kinase A
PMSF	phenylmethylsulphonyl fluoride
qPCR	quantitative polymerase chain reaction
RNA-seq	RNA sequencing
ROS	reactive oxygen species
SP	Spirulina peptides
TNF	tumor necrosis factor
TRP-1	tyrosinase-related protein 1
TRP-2	tyrosinase-related protein 2
TYR	tyrosinase
UV	ultraviolet
UVB	ultraviolet B

## References

[1] Logesh R., Prasad S.R., Chipurupalli S., Robinson N., Mohankumar S.K. (2023). Natural tyrosinase enzyme inhibitors: A path from melanin to melanoma and its reported pharmacological activities. Biochim. Biophys. Acta (BBA) Rev. Cancer.

[2] Figon F., Hurbain I., Heiligenstein X., Trépout S., Lanoue A., Medjoubi K., Somogyi A., Delevoye C., Raposo G., Casas J. (2021). Catabolism of lysosome-related organelles in color-changing spiders supports intracellular turnover of pigments. Proc. Natl. Acad. Sci. USA.

[3] Li S., Jang G.-B., Quach C., Liang C. (2019). Darkening with UVRAG. Autophagy.

[4] Kim J.-H., Hong A.-R., Kim Y.-H., Yoo H., Kang S.-W., Chang S.E., Song Y. (2020). JNK suppresses melanogenesis by interfering with CREB-regulated transcription coactivator 3-dependent MITF expression. Theranostics.

[5] Zhou S., Zeng H., Huang J., Lei L., Tong X., Li S., Zhou Y., Guo H., Khan M., Luo L. (2021). Epigenetic regulation of melanogenesis. Ageing Res. Rev..

[6] Sim Y.Y., Tan C.P., Cheong L.Z., Nyam K.L. (2022). *Hibiscus cannabinus* L. leaf and seed in cosmetic formulation: An integrated approach as antioxidant and melanogenesis inhibitor. Sustain. Mater. Technol..

[7] Lee K.W., Cho Y.-Y., Kim K.D. (2024). RCHY1 and OPTN: An E3-ligase and an autophagy receptor required for melanophagy, respectively. Autophagy.

[8] Wang X., Chen Y., Lan B., Wang Y., Lin W., Jiang X., Ye J., Shang B., Feng C., Liu J. (2022). Heterogeneity of tyrosine-based melanin anabolism regulates pulmonary and cerebral organotropic colonization microenvironment of melanoma cells. Theranostics.

[9] Lee J.H., An H., Kwon H., Lee S.-J., Park Y.H., Hwang J.S., Kim M.Y., Hwang H., Kim J.Y., Lee S.J. (2024). Engineering small-molecule analogues of altiratinib via CREB-regulated transcription co-activator 3-target screening for the development of potent and safe topical therapeutics against skin hyperpigmentary diseases. Clin. Transl. Med..

[10] Zhao M., Hong X., Abdullah, Yao R., Xiao Y. (2021). Rapid biosynthesis of phenolic glycosides and their derivatives from biomass-derived hydroxycinnamates. Green Chem..

[11] Xu T., Gu Z., Cheng L., Li C., Li Z., Hong Y. (2023). Stability, oxidizability, and topical delivery of resveratrol encapsulated in octenyl succinic anhydride starch/chitosan complex-stabilized high internal phase Pickering emulsions. Carbohydr. Polym..

[12] Victorelli F.D., Rodero C.F., Lutz-Bueno V., Chorilli M., Mezzenga R. (2023). Amyloid Fibrils Enhance the Topical Bio-Adhesivity of Liquid Crystalline Mesophase-Based Drug Formulations. Adv. Healthc. Mater..

[13] Zhao Q., Gu N., Li Y., Wu X., Ouyang Q., Deng L., Ma H., Zhu Y., Fang F., Ye H. (2024). Self-assembled gel microneedle formed by MS deep eutectic solvent as a transdermal delivery system for hyperpigmentation treatment. Mater. Today Bio.

[14] Boo Y.C. (2019). Human Skin Lightening Efficacy of Resveratrol and Its Analogs: From in Vitro Studies to Cosmetic Applications. Antioxidants.

[15] Ju X., Cheng S., Li H., Xu X., Wang Z., Du M. (2022). Tyrosinase inhibitory effects of the peptides from fish scale with the metal copper ions chelating ability. Food Chem..

[16] Boo Y.C. (2020). Up-or Downregulation of Melanin Synthesis Using Amino Acids, Peptides, and Their Analogs. Biomedicines.

[17] Zhu Y., Wang K., Jia X., Fu C., Yu H., Wang Y. (2024). Antioxidant peptides, the guardian of life from oxidative stress. Med. Res. Rev..

[18] Chataigner M., Mortessagne P., Lucas C., Pallet V., Layé S., Mehaignerie A., Bouvret E., Dinel A.L., Joffre C. (2021). Dietary fish hydrolysate supplementation containing n-3 LC-PUFAs and peptides prevents short-term memory and stress response deficits in aged mice. Brain Behav. Immun..

[19] Luo Q., Gao Z., Bai L., Ye H., Ye H., Wang Y., Gao Y., Chen T., Chen H., Liu Y. (2025). Bioactive Peptide-Based Composite Hydrogel for Myocardial Infarction Treatment: ROS Scavenging and Angiogenesis Regulation. Acta Biomater..

[20] Han J.H., Bang J.S., Choi Y.J., Choung S.Y. (2019). Oral administration of oyster (*Crassostrea gigas*) hydrolysates protects against wrinkle formation by regulating the MAPK pathway in UVB-irradiated hairless mice. Photochem. Photobiol. Sci..

[21] Peng Z., Gao J., Su W., Cao W., Zhu G., Qin X., Zhang C., Qi Y. (2022). Purification and Identification of Peptides from Oyster (*Crassostrea hongkongensis*) Protein Enzymatic Hydrolysates and Their Anti-Skin Photoaging Effects on UVB-Irradiated HaCaT Cells. Mar. Drugs.

[22] Richter M., Lalli E., Ruggiero C. (2023). Complex and pleiotropic signaling pathways regulated by the secreted protein augurin. Cell Commun. Signal..

[23] Wu L.-C., Lin Y.-Y., Yang S.-Y., Weng Y.-T., Tsai Y.-T. (2011). Antimelanogenic effect of c-phycocyanin through modulation of tyrosinase expression by upregulation of ERK and downregulation of p38 MAPK signaling pathways. J. Biomed. Sci..

[24] Bortolini D.G., Maciel G.M., Fernandes I.d.A.A., Pedro A.C., Rubio F.T.V., Branco I.G., Haminiuk C.W.I. (2022). Functional properties of bioactive compounds from *Spirulina* spp.: Current status and future trends. Food Chem. Mol. Sci..

[25] Lafarga T., Fernández-Sevilla J.M., González-López C., Acién-Fernández F.G. (2020). Spirulina for the food and functional food industries. Food Res. Int..

[26] Xie M., Jiang Z., Lin X., Wei X. (2024). Application of plant extracts cosmetics in the field of anti-aging. J. Dermatol. Sci. Cosmet. Technol..

[27] Yang Q., Hu Z., Jiang H., Wang J., Han H., Shi W., Qian H. (2025). Recent advances, strategies, and future perspectives of peptide-based drugs in clinical applications. Chin. J. Nat. Med..

[28] Delk S.C., Chattopadhyay A., Escola-Gil J.C., Fogelman A.M., Reddy S.T. (2021). Apolipoprotein mimetics in cancer. Semin. Cancer Biol..

[29] Bojarska J. (2022). Advances in Research of Short Peptides. Molecules.

[30] Liu P., Lee M.K., Choi J.W., Choi Y.H., Nam T.J. (2019). Crude protein from spirulina increases the viability of CCD-986sk cells via the EGFR/MAPK signaling pathway. Int. J. Mol. Med..

[31] Kose A., Oncel S.S. (2022). Design of melanogenesis regulatory peptides derived from phycocyanin of the microalgae Spirulina platensis. Peptides.

[32] Masuda T., Odaka Y., Ogawa N., Nakamoto K., Kuninaga H. (2008). Identification of Geranic Acid, a Tyrosinase Inhibitor in Lemongrass (*Cymbopogon citratus*). J. Agric. Food Chem..

[33] Nihei K.-i., Kubo I. (2019). Benzonitriles as tyrosinase inhibitors with hyperbolic inhibition manner. Int. J. Biol. Macromol..

[34] Kubglomsong S., Theerakulkait C., Reed R.L., Yang L., Maier C.S., Stevens J.F. (2018). Isolation and Identification of Tyrosinase-Inhibitory and Copper-Chelating Peptides from Hydrolyzed Rice-Bran-Derived Albumin. J. Agric. Food Chem..

[35] Ma Y., Zhang D., Liu M., Li Y., Lv R., Li X., Wang Q., Ren D., Wu L., Zhou H. (2022). Identification of Antioxidant Peptides Derived from Tilapia (*Oreochromis niloticus*) Skin and Their Mechanism of Action by Molecular Docking. Foods.

[36] Zhang L., Zhu M.-f., Tu Z.-c., Zhao Y., Wang H., Li G.-j., Wang H., Sha X.-m. (2017). *a*-Glucosidase inhibition, anti-glycation and antioxidant activities of *Liquidambar formosana* Hance leaf, and identification of phytochemical profile. S. Afr. J. Bot..

[37] Ha A.T., Rahmawati L., You L., Hossain M.A., Kim J.-H., Cho J.Y. (2022). Anti-Inflammatory, Antioxidant, Moisturizing, and Antimelanogenesis Effects of Quercetin 3-O-β-D-Glucuronide in Human Keratinocytes and Melanoma Cells via Activation of NF-κB and AP-1 Pathways. Int. J. Mol. Sci..

[38] Lee R., Ko H.J., Kim K., Sohn Y., Min S.Y., Kim J.A., Na D., Yeon J.H. (2020). Anti-melanogenic effects of extracellular vesicles derived from plant leaves and stems in mouse melanoma cells and human healthy skin. J. Extracell. Vesicles.

[39] Li Z., Wang Z., Zhou Q., Wang R., Xiong Z., Wu Y., Li Y., Liu L., Jiang C., Zhu H. (2024). The molecular mechanisms underlying optical isomer arbutin permeating the skin: The molecular interaction between arbutin and skin components. Int. J. Pharm..

[40] Han J.-H., Bang J.S., Choi Y.J., Choung S.-Y. (2019). Anti-melanogenic effects of oyster hydrolysate in UVB-irradiated C57BL/6J mice and B16F10 melanoma cells via downregulation of cAMP signaling pathway. J. Ethnopharmacol..

[41] Chen S., Zhou Y., Chen Y., Gu J. (2018). fastp: An ultra-fast all-in-one FASTQ preprocessor. Bioinformatics.

[42] Li B., Dewey C.N. (2011). RSEM: Accurate transcript quantification from RNA-Seq data with or without a reference genome. BMC Bioinform..

[43] Ashburner M., Ball C.A., Blake J.A., Botstein D., Butler H., Cherry J.M., Davis A.P., Dolinski K., Dwight S.S., Eppig J.T. (2000). Gene Ontology: Tool for the unification of biology. Nat. Genet..

[44] Kanehisa M., Goto S. (2000). KEGG: Kyoto Encyclopedia of Genes and Genomes. Nucleic Acids Res..

[45] Hirobe T. (1995). Structure and function of melanocytes: Microscopic morphology and cell biology of mouse melanocytes in the epidermis and hair follicle. Histol. Histopathol..

[46] Müller-Röver S., Foitzik K., Paus R., Handjiski B., van der Veen C., Eichmüller S., McKay I.A., Stenn K.S. (2001). A comprehensive guide for the accurate classification of murine hair follicles in distinct hair cycle stages. J. Investig. Dermatol..

[47] Lee T.H., Seo J.O., Do M.H., Ji E., Baek S.-H., Kim S.Y. (2014). Resveratrol-Enriched Rice Down-Regulates Melanin Synthesis in UVB-Induced Guinea Pigs Epidermal Skin Tissue. Biomol. Ther..

[48] Park S.H., Jo Y.-J. (2019). Static hydrothermal processing and fractionation for production of a collagen peptide with anti-oxidative and anti-aging properties. Process Biochem..

[49] Schurink M., van Berkel W.J.H., Wichers H.J., Boeriu C.G. (2007). Novel peptides with tyrosinase inhibitory activity. Peptides.

[50] Pavan M.E., López N.I., Pettinari M.J. (2020). Melanin biosynthesis in bacteria, regulation and production perspectives. Appl. Microbiol. Biotechnol..

[51] Wang W., Gao Y., Wang W.W., Zhang J.Y., Yin J.F., Le T., Xue J.J., Engelhardt U.H., Jiang H.Y. (2022). Kojic Acid Showed Consistent Inhibitory Activity on Tyrosinase from Mushroom and in Cultured B16F10 Cells Compared with Arbutins. Antioxidants.

[52] Boo Y.C. (2021). Arbutin as a Skin Depigmenting Agent with Antimelanogenic and Antioxidant Properties. Antioxidants.

[53] Meneses-Gutiérrez C.L., Hernández-Damián J., Pedraza-Chaverri J., Guerrero-Legarreta I., Téllez D.I., Jaramillo-Flores M.E. (2019). Antioxidant Capacity and Cytotoxic Effects of Catechins and Resveratrol Oligomers Produced by Enzymatic Oxidation against T24 Human Urinary Bladder Cancer Cells. Antioxidants.

[54] Lazinski L.M., Beaumet M., Roulier B., Gay R., Royal G., Maresca M., Haudecoeur R. (2024). Design and synthesis of 4-amino-2′,4′-dihydroxyindanone derivatives as potent inhibitors of tyrosinase and melanin biosynthesis in human melanoma cells. Eur. J. Med. Chem..

[55] Zolghadri S., Beygi M., Mohammad T.F., Alijanianzadeh M., Pillaiyar T., Garcia-Molina P., Garcia-Canovas F., Munoz-Munoz J., Saboury A.A. (2023). Targeting tyrosinase in hyperpigmentation: Current status, limitations and future promises. Biochem. Pharmacol..

[56] Segel I.H. (1975). Enzyme Kinetics: Behavior and Analysis of Rapid Equilibrium and Steady State Enzyme Systems.

[57] Wijaya C., Elya B., Yanuar A. (2018). Study of tyrosinase inhibitory activity and phytochemical screening of *Cassia fistula* L. leave. Int. J. Appl. Pharm..

[58] Zhang J., Wang C., Wang C., Sun B., Qi C. (2018). Understanding the role of extracts from sea buckthorn seed residues in anti-melanogenesis properties on B16F10 melanoma cells. Food Funct..

[59] Tadesse S.A., Emire S.A. (2020). Production and processing of antioxidant bioactive peptides: A driving force for the functional food market. Heliyon.

[60] Mirzaei M., Mirdamadi S., Safavi M., Soleymanzadeh N. (2020). The stability of antioxidant and ACE-inhibitory peptides as influenced by peptide sequences. LWT.

[61] Sae-Leaw T., Karnjanapratum S., O’Callaghan Y.C., O’Keeffe M.B., FitzGerald R.J., O’Brien N.M., Benjakul S. (2017). Purification and identification of antioxidant peptides from gelatin hydrolysate of seabass skin. J. Food Biochem..

[62] Chen H., Wang S., Zhou A., Miao J., Liu J., Benjakul S. (2020). A novel antioxidant peptide purified from defatted round scad (*Decapterus maruadsi*) protein hydrolysate extends lifespan in Caenorhabditis elegans. J. Funct. Foods.

[63] Jang H.L., Liceaga A.M., Yoon K.Y. (2016). Purification, characterisation and stability of an antioxidant peptide derived from sandfish (*Arctoscopus japonicus*) protein hydrolysates. J. Funct. Foods.

[64] Wu R., Huang J., Huan R., Chen L., Yi C., Liu D., Wang M., Liu C., He H. (2021). New insights into the structure-activity relationships of antioxidative peptide PMRGGGGYHY. Food Chem..

[65] Lee E.J., Kim J.Y., Oh S.H. (2016). Advanced glycation end products (AGEs) promote melanogenesis through receptor for AGEs. Sci. Rep..

[66] Tsang T.-F., Chan B., Tai W.C.-S., Huang G., Wang J., Li X., Jiang Z.H., Hsiao W.L.W. (2019). Gynostemma pentaphyllum saponins induce melanogenesis and activate cAMP/PKA and Wnt/β-catenin signaling pathways. Phytomedicine.

[67] Pillaiyar T., Manickam M., Namasivayam V. (2017). Skin whitening agents: Medicinal chemistry perspective of tyrosinase inhibitors. J. Enzym. Inhib. Med. Chem..

[68] Yang S.H., Tsatsakis A.M., Tzanakakis G., Kim H.S., Le B., Sifaki M., Spandidos D.A., Tsukamoto C., Golokhvast K.S., Izotov B.N. (2017). Soyasaponin Ag inhibits α-MSH-induced melanogenesis in B16F10 melanoma cells via the downregulation of TRP-2. Int. J. Mol. Med..

[69] Chen Y.-M., Su W.-C., Li C., Shi Y., Chen Q.-X., Zheng J., Tang D.-L., Chen S.-M., Wang Q. (2019). Anti-melanogenesis of novel kojic acid derivatives in B16F10 cells and zebrafish. Int. J. Biol. Macromol..

[70] Chan C.-F., Huang C.-C., Lee M.-Y., Lin Y.-S. (2014). Fermented Broth in Tyrosinase- and Melanogenesis Inhibition. Molecules.

[71] Aguilar-Toalá J., Hernández-Mendoza A., González-Córdova A., Vallejo-Cordoba B., Liceaga A. (2019). Potential role of natural bioactive peptides for development of cosmeceutical skin products. Peptides.

[72] Lee H.-R., Jung J.M., Seo J.-Y., Chang S.E., Song Y. (2021). Anti-melanogenic property of ginsenoside Rf from Panax ginseng via inhibition of CREB/MITF pathway in melanocytes and ex vivo human skin. J. Ginseng Res..

[73] Han C., Lin B., Huang X., Mao Z., Kong X., Fang L., Xue P., Wang A., Zhang F. (2023). Quinoa husk peptides reduce melanin content via Akt signaling and apoptosis pathways. iScience.

[74] Li H.-L., Li M.-J., Xiong G.-Q., Cai J., Liao T., Zu X.-Y. (2023). Silver Carp (*Hypophthalmichthys molitrix*) Scale Collagen Peptides-1 (SCPs1) Inhibit Melanogenesis through Downregulation of the cAMP-CREB Signaling Pathway. Nutrients.

[75] Yoon J.H., Jang W.Y., Park S.H., Kim H.G., Shim Y.Y., Reaney M.J.T., Cho J.Y. (2023). Anti-Melanogenesis Effects of a Cyclic Peptide Derived from Flaxseed via Inhibition of CREB Pathway. Int. J. Mol. Sci..

[76] Oka M., Nagai H., Ando H., Fukunaga M., Matsumura M., Araki K., Ogawa W., Miki T., Sakaue M., Tsukamoto K. (2000). Regulation of Melanogenesis through Phosphatidylinositol 3-Kinase-Akt Pathway in Human G361 Melanoma Cells. J. Investig. Dermatol..

[77] Choi H., Yoon J.-H., Youn K., Jun M. (2022). Decursin prevents melanogenesis by suppressing MITF expression through the regulation of PKA/CREB, MAPKs, and PI3K/Akt/GSK-3β cascades. Biomed. Pharmacother..

[78] Chen W., Hashimoto K., Omata Y., Ohgami N., Tazaki A., Deng Y., Kondo-Ida L., Intoh A., Kato M. (2019). Adsorption of molybdenum by melanin. Environ. Health Prev. Med..

[79] You Y.-J., Wu P.-Y., Liu Y.-J., Hou C.-W., Wu C.-S., Wen K.-C., Lin C.-Y., Chiang H.-M. (2019). Sesamol Inhibited Ultraviolet Radiation-Induced Hyperpigmentation and Damage in C57BL/6 Mouse Skin. Antioxidants.

[80] Szepietowski J.C., Krajewski P.K., Pacan P. (2024). Psoriasis: An inflammatory skin disease affecting the mind. J. Eur. Acad. Dermatol. Venereol..

[81] Tweedell R.E., Kanneganti T.-D. (2023). NLRP12-PANoptosome in haemolytic, infectious and inflammatory diseases. Clin. Transl. Med..

[82] Martin S.F. (2012). Contact dermatitis: From pathomechanisms to immunotoxicology. Exp. Dermatol..

[83] Miller L.S., Cho J.S. (2011). Immunity against Staphylococcus aureus cutaneous infections. Nat. Rev. Immunol..

[84] Behrends U.t.a., Eißner G., Bornkamm G.W., Peter R.U., Hintermeier-Knabe R., Holler E., Caughman S.W., Degitz K. (1994). Ionizing Radiation Induces Human Intercellular Adhesion Molecule-1 In Vitro. J. Investig. Dermatol..

[85] Maghfour J., Olayinka J., Hamzavi I.H., Mohammad T.F. (2022). A Focused review on the pathophysiology of post-inflammatory hyperpigmentation. Pigment. Cell Melanoma Res..

[86] Kim H.M., Oh S., Yoon J.H., Kang D., Son M., Byun K. (2021). Radiofrequency Irradiation Attenuates High-Mobility Group Box 1 and Toll-like Receptor Activation in Ultraviolet B–Induced Skin Inflammation. Molecules.

[87] Fu C., Chen J., Lu J., Yi L., Tong X., Kang L., Pei S., Ouyang Y., Jiang L., Ding Y. (2020). Roles of inflammation factors in melanogenesis (Review). Mol. Med. Rep..

[88] Zhou J., Ling J., Wang Y., Shang J., Ping F. (2016). Cross-talk between interferon-gamma and interleukin-18 in melanogenesis. J. Photochem. Photobiol. B Biol..

[89] Zhou J., Song J., Ping F., Shang J. (2014). Enhancement of the p38 MAPK and PKA signaling pathways is associated with the pro-melanogenic activity of Interleukin 33 in primary melanocytes. J. Dermatol. Sci..

[90] Scott G., Jacobs S., Leopardi S., Anthony F.A., Learn D., Malaviya R., Pentland A. (2005). Effects of PGF2α on human melanocytes and regulation of the FP receptor by ultraviolet radiation. Exp. Cell Res..

[91] Mann T., Gerwat W., Batzer J., Eggers K., Scherner C., Wenck H., Stäb F., Hearing V.J., Röhm K.H., Kolbe L. (2018). Inhibition of Human Tyrosinase Requires Molecular Motifs Distinctively Different from Mushroom Tyrosinase. J. Investig. Dermatol..

