# Supplementary material for: Spirulina Peptides Suppress UVB-Induced Skin Hyperpigmentation via Integrated Modulation of Melanogenesis and Inflammatory Pathways
Source: Antioxidants (Basel). 2026 Jan 30;15(2):181. doi: 10.3390/antiox15020181 (PMC12937610; doi:10.3390/antiox15020181)
Supplement: Supplementary file 1 [file antioxidants-15-00181-s001.zip › antioxidants-4079342-supplementary.pdf]

Spirulina Peptides Suppress UVB-Induced Skin Hyperpigmentation via Integrated Modulation of Melanogenesis and Inflammatory Pathways

Qiying Zeng [1,#], Kaiye Yang [2,#], Hongtao Gu [1], Changzhi Dong [3], Wei Zhou [1,*], Zhiyun Du [1,*]

Co-first author: Qiying Zeng, e-mail: 13826020732@163.com; Kaiye Yang, e-mail: kyle.yang@infinitus-int.com

Corresponding authors: zhou_wei@gdut.edu.cn (W.Z.), and zhiyundu@gdut.edu.cn (Z.D.).

[1] School of Biomedical and Pharmaceutical Sciences, Guangdong University of Technology, Guangzhou 510006, China

[2] Infinitus (China) Company Ltd., Guangzhou 510405, China

[3] Université Paris Cité, CNRS UMR 8251, INSERM ERL 1133, Unité de Biologie Fonctionnelle et Adaptative F-75013, Paris, France

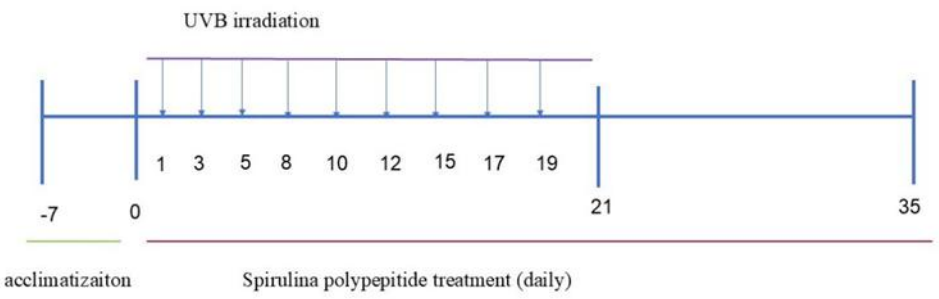

Supplementary Figure 1. Process of the animal experiment.

Supplementary Table 1. The composition of hydrolyzed amino acids in SP

Amino acids	Content (g/100g)
Asparagine	7.4833
Serine	3.1561
Glycine	3.6153
Cysteine	0.3267
Methionine	1.0999
Leucine	5.5725
Phenylalanine	1.9644
Histidine	1.0059
Proline	3.9853
Threonine	3.6091
Glutamic acid	13.4346
Alanine	8.7136
Valine	4.6771
Isoleucine	4.0202
Tyrosine	1.6945
Lysine	3.8120
Arginine	4.5565

Total	72.7270

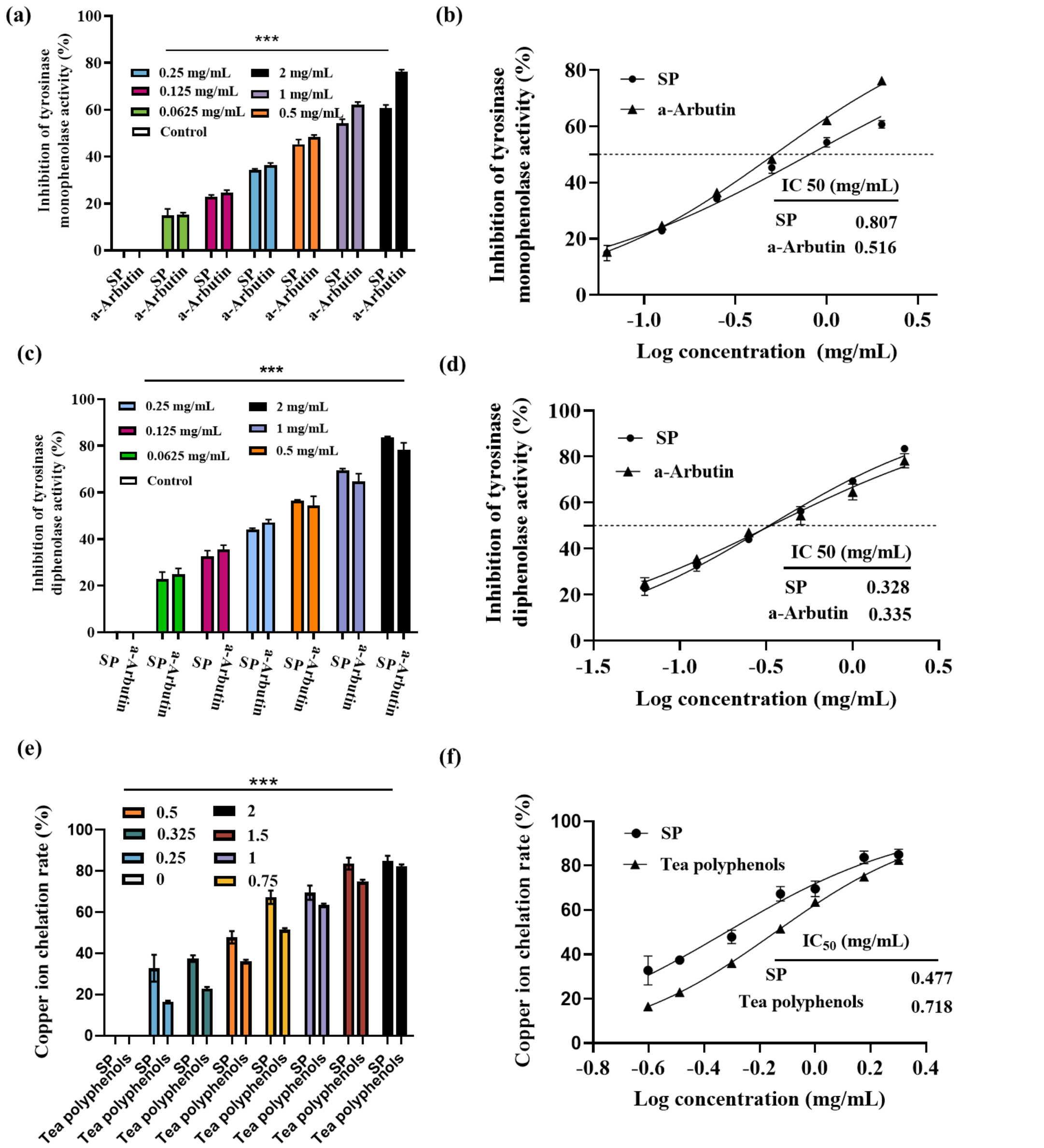

Supplementary Figure 2. Tyrosinase inhibition and copper chelation by SP. (a) Monophenolase inhibition (%) and (b) corresponding IC50 curves. (c) Diphenolase inhibition (%) and (d) IC_{50} curves. (e) Copper ion chelation activity (%) and (f) IC_{50} curves. a-Arbutin and Tea polyphenols served as positive controls. Statistical results are expressed as the means ± SD of three independent experiments. *** indicate $p < 0.001$ compared with the control.

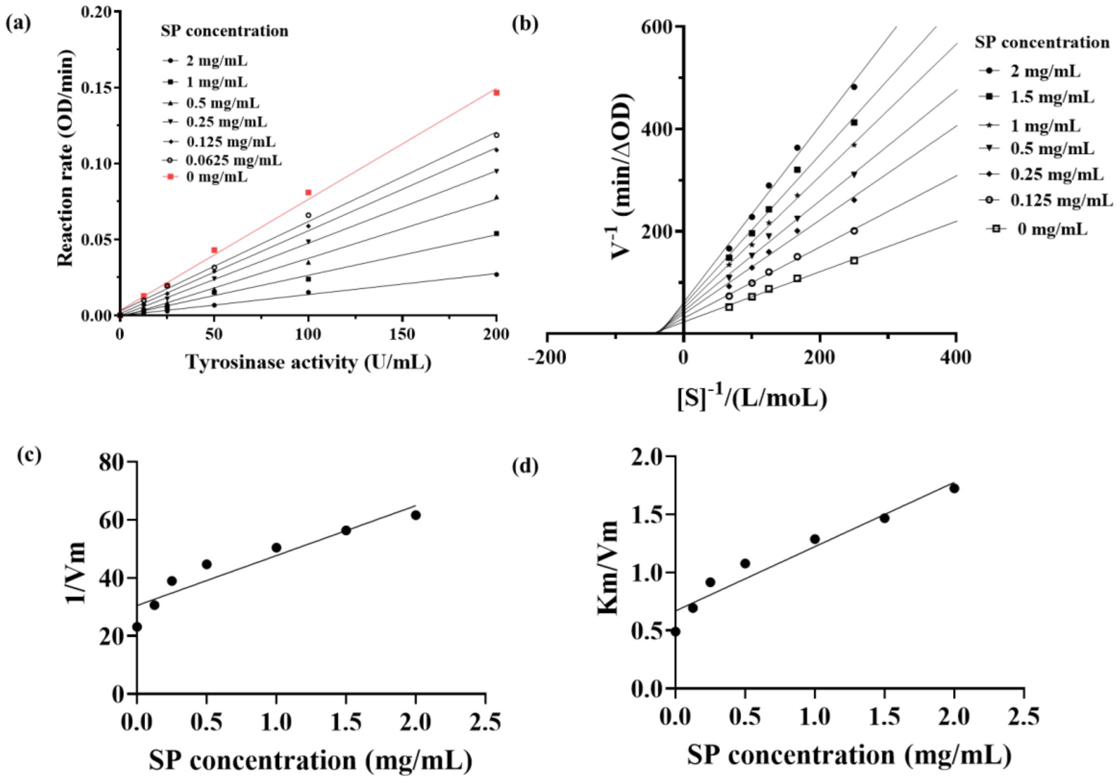

Supplementary Figure 3. (a) Inhibitory mechanism of SP on TYR; (b) Lineweaver–Burk plots for inhibition of TYR in the presence of SP; The plot of the slope (c) and intercept (d) versus SP concentrations to determine the inhibition constant.

Supplementary Table 2. Kinetic parameters of TYR inhibition

SP concentration	Regression equation	V_m (ΔOD/min)	K_m (mol/L)	K_i (mg/mL)	K_{is} (mg/mL)
2 mg/mL	Y = 1.724*X + 61.66	0.0162	0.0280		
1.5 mg/mL	Y = 1.467*X + 56.38	0.0177	0.0260		
1 mg/mL	Y = 1.287*X + 50.43	0.0198	0.0255		
0.5 mg/mL	Y = 1.077*X + 44.68	0.0224	0.0241	2.68	3.05
0.25 mg/mL	Y = 0.9166*X + 38.98	0.0257	0.0235		
0.125 mg/mL	Y = 0.6944*X + 30.65	0.0326	0.0227		
0 mg/mL	Y = 0.4908*X + 23.17	0.0432	0.0212		

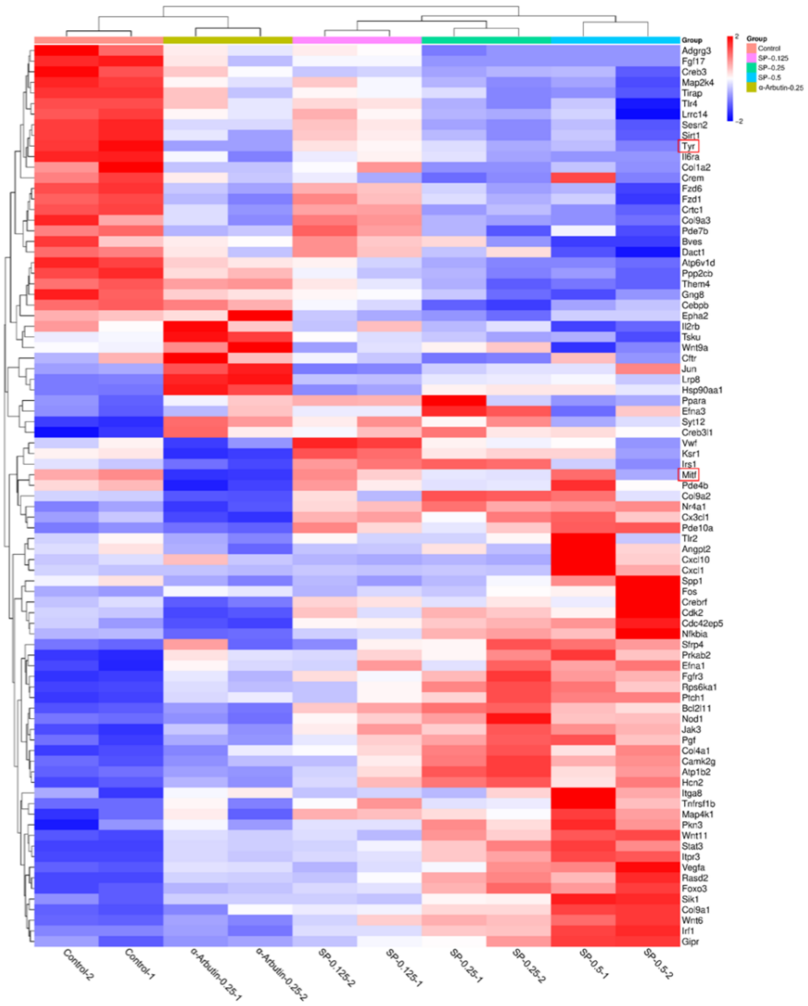

Supplementary Figure 4. Distinct gene expression profiles revealed by heatmap analysis. Hierarchical clustering of samples (Control, α-Arbutin-0.25 mg/ml, SP-0.125, SP-0.25, SP-0.5 mg/mL) and differentially expressed genes demonstrate distinct transcriptional patterns induced by SP treatment. Rows represent individual DEGs, and columns represent biological replicates for each treatment group. Gene expression levels are represented by color intensity based on row Z-scores, with red indicating higher expression and blue indicating lower expression relative to the mean expression across all samples for that gene.

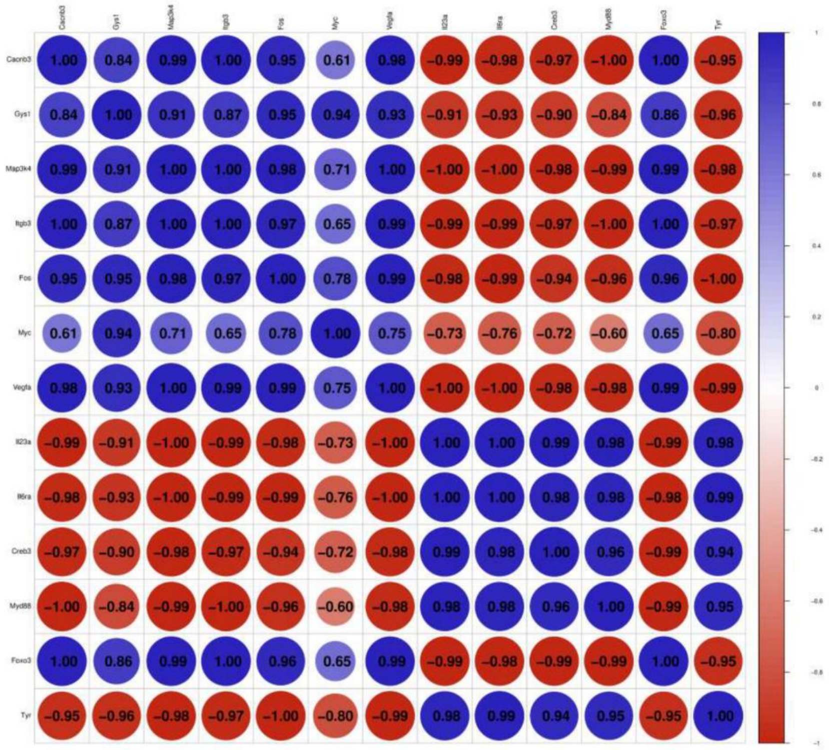

Supplementary Figure 5. Correlation matrix heatmap visualizing co-expression patterns among melanogenesis-related DEGs. Significant Pearson correlations ($|r| > 0.6$, FDR-adjusted $p < 0.05$) are shown between DEGs (identified in SP 0.5 mg/mL vs. Control; $|log2FC| \geq 1$, FDR < 0.05) associated with MAPK, JAK-STAT, PI3K/AKT, and cAMP pathways. Colors indicate correlation strength and direction (Dark Red: strong positive; Dark Blue: strong negative).
